# Clinical efficacy and multi-target mechanisms of liver-dispersing, bile-inducing, and heat-clearing TCM formulas in cholelithiasis: network meta-analysis, network pharmacology, and molecular docking study

**DOI:** 10.3389/fmed.2026.1944318

**Published:** 2026-10-07

**Authors:** Lian Cai, Yueqiao Lyu, Peipei Li, Mingzhu Li

**Affiliations:** 1 School of Traditional Chinese Medicine, Anhui University of Chinese Medicine, Hefei, China; 2 Dongzhimen Hospital of Beijing University of Chinese Medicine, Beijing, China

**Keywords:** cholelithiasis, molecular docking, network meta-analysis, network pharmacology, traditional Chinese medicine

## Abstract

**Objective:**

Although liver-soothing, gallbladder-benefiting, and heat-clearing (SGLDQR) traditional Chinese medicine (TCM) formulas are widely used for cholelithiasis, their systematic efficacy and molecular mechanisms remain poorly understood. This study integrated network meta-analysis (NMA), data mining, network pharmacology, and molecular docking to evaluate the clinical efficacy and clarify the molecular mechanisms of SGLDQR TCM formulas against cholelithiasis.

**Methods:**

Randomized controlled trials (RCTs) up to December 2024 were retrieved from eight databases. Risk of bias was evaluated using Cochrane RoB 2.0. NMA was conducted using Stata (v14.0) and R (v4.4.3), with treatments ranked by the surface under the cumulative ranking curve (SUCRA) and evidence certainty evaluated via CINeMA. Prescription patterns were mined using the Apriori algorithm. SGLDQR–cholelithiasis common targets were identified across multiple databases, followed by hub target screening using degree centrality and maximal clique centrality (MCC) algorithms and Gene Ontology (GO) and Kyoto Encyclopedia of Genes and Genomes (KEGG) functional enrichment. Finally, blind whole-protein molecular docking of the top three hub targets with active ingredients was performed using AutoDock Vina (v1.1.2) and verified via PLIP.

**Results:**

Thirteen RCTs involving 1,364 patients and 12 SGLDQR formulas were included. NMA revealed that herbal formulas outperformed conventional Western medicine. Self-prepared Danshi Decoction (ZNDST) ranked highest in improving total effective rate (SUCRA: 88.2%), the Shugan Lidan Qingre formula (SGLDQRF) demonstrated potential superiority in improving TCM syndrome scores (SUCRA: 85.7%), and Rongpai Danshi Capsules (RPDSJN) exhibited a modulatory effect in reducing serum AST (99.9%) and ALT (100.0%) levels. Adverse events were mild and self-limiting. CINeMA certainty of evidence was low to very low. Data mining indicated that these formulas were predominantly cold in nature, combined bitter, pungent, and sweet flavors, and mainly targeted the spleen, liver, and stomach meridians, focusing on clearing heat, draining dampness, soothing the liver, and promoting gallbladder function, driven by 8 core herbs. Network pharmacology identified active compounds such as quercetin, wogonin, paeoniflorin, and aloe-emodin, which acted on key targets including IL-6, IL-1β, and TNF-α, primarily enriched in inflammatory cascades and lipid-metabolism pathways. Molecular docking predicted favorable binding affinities between the key active compounds and core targets.

**Conclusion:**

SGLDQR TCM formulas demonstrate potential comprehensive clinical benefits in the conservative management of cholelithiasis. However, constrained by the evidence network geometry and study heterogeneity, the ranking results and syndrome-differentiation strategies remain exploratory. The predicted synergism between anti-inflammatory and metabolic pathways provides a mechanistic hypothesis for subsequent research. Future rigorous multicenter, double-blind RCTs along with *in vitro* and *in vivo* functional assays are warranted for validation.

**Systematic Review Registration:**

https://www.crd.york.ac.uk/PROSPERO/, identifier CRD42024598181.

## 1 Introduction

Cholelithiasis is a prevalent biliary disorder with a 6.1% global prevalence (1, 2), posing substantial risks of acute cholecystitis, pancreatitis, and gastrointestinal malignancies (3–5). Although laparoscopic cholecystectomy remains the gold standard, its perioperative risks and long-term safety controversies underscore the need for effective non-surgical alternatives (6). In Traditional Chinese Medicine (TCM), the core pathogenesis of cholelithiasis centers on liver-gallbladder qi stagnation, which induces damp-heat and stasis, impeding bile secretion and precipitating the formation of calculi (7–9). Accordingly, liver-dispersing, bile-inducing, and heat-clearing (SGLDQR) formulas are widely applied in clinical practice. However, TCM interventions face dual bottlenecks: a lack of high-quality multicenter evidence and unclarified “multi-component, multi-target” molecular mechanisms. To address these gaps, this study establishes a multiscale integrated framework spanning “clinical efficacy evaluation, herb-compatibility deciphering, and mechanism verification.” By sequentially employing network meta-analysis (NMA), association rule mining, network pharmacology, and molecular docking, we systematically elucidate the therapeutic material basis and regulatory networks of SGLDQR formulas. This work aims to provide high-level, evidence-based validation and theoretical support for precise, integrated traditional Chinese and Western medicine interventions for cholelithiasis.

## 2 Materials and methods

### 2.1 Study registration and design

This systematic review and NMA strictly adhered to the PRISMA guidelines (10). The protocol of this study was registered in PROSPERO (CRD42024598181).

### 2.2 Inclusion and exclusion criteria

Randomized controlled trials (RCTs) in any language were included if they explicitly detailed randomization methods and provided complete statistical reporting, raw data, and outcome measures. Eligibility criteria were defined according to the PICOS framework: (1) participants comprised unoperated patients with cholelithiasis diagnosed via original trial-specific criteria (principally ultrasonography), with diagnostic consistency verified against the *Expert Consensus on Integrated Traditional Chinese and Western Medicine Diagnosis and Treatment of Cholelithiasis (2025)* (11) solely to ensure clinical equivalence rather than retrospectively alter eligibility; (2) interventions evaluated were TCM formulas with SGLDQR properties (decoctions, granules, capsules, or proprietary Chinese medicines); and (3) comparators were conventional Western medicine (WM) active controls. Specifically, biliary agents with established litholytic or choleretic actions—such as ursodeoxycholic acid (UDCA), chenodeoxycholic acid (CDCA), and anethole trithione (ANT)—were consolidated into a single WM node owing to their shared therapeutic goal of calculus dissolution and expulsion.

Studies were excluded if they involved: (1) duplicate publications or overlapping data; (2) patients without cholelithiasis or with prior biliary tract surgery; (3) control groups receiving non-Western medicine therapies or intervention groups treated with multi-formula TCM combinations instead of a single SGLDQR prescription; (4) trials lacking essential outcome data that could not be retrieved after contacting the authors; or (5) reviews, animal studies, conference abstracts, and patents.

### 2.3 Outcomes

The primary outcome was the overall clinical efficacy, defined as the percentage of cases that achieved recovery, marked effect, or improvement, and was calculated as follows: (Number of Effective Cases/Total Number of Cases) × 100%. Secondary outcomes included: (1) TCM syndrome scores; (2) serum aspartate aminotransferase (AST) levels; (3) serum alanine aminotransferase (ALT) levels; and (4) adverse events.

### 2.4 Literature search strategy

A comprehensive literature search was conducted across eight databases, including PubMed, Embase, The Cochrane Library, Web of Science, CNKI, WanFang, VIP, and CBM, from their inception dates up to December 31, 2024. The search strategy utilized a combination of Medical Subject Headings (MeSH) terms and free-text keywords related to cholelithiasis, TCM, and RCTs. The detailed PubMed search strategy is presented in Table 1, and the search strategies for the remaining databases are provided in Supplementary Information S1.

**Table 1:** Search strategy employed in the pubMed database.

Database	D	Search Strategy
PubMed	#1	“Cholelithiasis”[Mesh] OR “Choledocholithiasis”[Mesh] OR Cholelithiases[Title/Abstract] OR “Gallstone Disease”[Title/Abstract] OR “Gallstone Diseases”[Title/Abstract] OR Cholelithiasis[Title/Abstract] OR Choledocholithiasis[Title/Abstract] OR “Common Bile Duct”[Title/Abstract]
	#2	“Medicine, Chinese Traditional”[Mesh] OR “Drugs, Chinese Herbal"[Mesh] OR “Chinese Medicine, Traditional”[Title/Abstract] OR “Chinese Traditional Medicine”[Title/Abstract] OR “Traditional Chinese Medicine”[Title/Abstract] OR TCM[Title/Abstract] OR “Chinese Drugs, Plant”[Title/Abstract] OR “Chinese Herbal Drugs”[Title/Abstract] OR “Plant Extracts, Chinese”[Title/Abstract] OR “Chinese Herbal”[Title/Abstract] OR Tang[Title/Abstract] OR Fang[Title/Abstract] OR Prescription[Title/Abstract]
	#3	“Randomized Controlled Trials as Topic”[Mesh] OR “Randomized Controlled Trial”[Publication Type] OR RCT[Title/Abstract] OR Randomly[Title/Abstract] OR Randomized[Title/Abstract] OR “Controlled Clinical Trial”[Title/Abstract] OR “Clinical Trial”[Title/Abstract] OR “Clinical Study”[Title/Abstract]
	#4	#1 AND #2 AND #3

### 2.5 Data extraction and risk of bias assessment

All retrieved records were imported into NoteExpress software for duplication identification and removal. Two reviewers independently screened the titles, abstracts, and full texts, with any disagreements resolved through consultation with a third reviewer. Data extraction encompassed the first author, publication year, sample size, diagnostic criteria, control regimens and dosages, treatment duration, baseline disease characteristics, clinical efficacy criteria, herbal formula compositions, and reported outcome measures, all of which were independently cross-checked by two reviewers. The risk of bias was assessed using the Cochrane Risk of Bias 2.0 (RoB 2.0) tool (12), which encompasses five distinct domains: bias arising from the randomization process, bias due to deviations from intended interventions, bias due to missing outcome data, bias in measurement of the outcome, and bias in selection of the reported result.

### 2.6 Statistical analysis

Pairwise meta-analyses were conducted using Stata (v14.0) (13). Relative risk (*RR*) was employed for dichotomous outcomes, while mean difference (*MD*) was utilized for continuous variables, both accompanied by their corresponding 95% confidence interval (95% *CI*). Heterogeneity was evaluated via the Cochrane *Q* test and the *I*^2^ statistic; a fixed-effects model was applied if *p* >= 0.05 and *I*^2^ < 50%, failing which a random-effects model was implemented. Potential sources of heterogeneity were explored using subgroup analyses and meta-regression, with estimation robustness confirmed via leave-one-out sensitivity analyses. NMA was performed using the network package in R (v4.4.3) to establish evidence networks and synthesise comparative effect sizes with 95% *CIs*. Sensitivity analyses were conducted to verify the robustness of pooled estimates against potential heterogeneity, whilst publication bias and small-study effects were appraised using comparison-adjusted funnel plots alongside Egger's linear regression test. Treatment hierarchies were determined via the surface under the cumulative ranking curve (SUCRA) derived from 100,000 Monte Carlo draws, wherein higher SUCRA values consistently denoted superior therapeutic efficacy across all outcomes (clinical efficacy, TCM syndrome scores, serum AST and ALT levels).

### 2.7 Assessment of certainty of evidence

The certainty of evidence for network estimates of total clinical efficacy was systematically evaluated using the Confidence In Network Meta-Analysis (CINeMA) framework (14) via its online platform (https://cinema.ispm.unibe.ch/). Assessments addressed six methodological domains: within-study bias, reporting bias, indirectness, imprecision, heterogeneity, and inconsistency, with each designated as having “no concerns”, “some concerns”, or “major concerns”. Certainty was initially set to “high” and systematically downgraded in accordance with identified risks. To prevent undue penalisation, correlated domains were appraised integratively rather than additively to avoid double-counting (for instance, where wide confidence or prediction intervals concurrently triggered flags for both heterogeneity and imprecision, or where clinical indirectness manifested directly as statistical inconsistency). The finalised certainty of evidence for each pairwise comparison was categorised as high, moderate, low, or very low.

### 2.8 Analysis of TCM prescription patterns

TCM formulas and botanical drugs extracted from the NMA were standardized for nomenclature and classified by therapeutic efficacy in accordance with the Pharmacopoeia of the People's Republic of China (2020 Edition) (15) and Traditional Chinese Medicine Philology (New Century 4th Edition) (16). Microsoft Excel was utilized to analyze the frequency, medicinal properties, and functional classifications of the botanical drugs. Furthermore, association rule mining based on the Apriori algorithm was executed using R (v4.4.3) to uncover core herbal combinations and prescription patterns (17).

### 2.9 Network pharmacology analysis

Core medicinals prioritised via usage frequency and association rule mining were subsequently screened based on database annotation feasibility. To safeguard analytical feasibility and disease-specific signal resolution in downstream network pharmacology, two methodological exclusion criteria were pre-specified: (1) animal-derived medicinal materials with insufficient small-molecule compound–target annotations in public databases; and (2) broad-spectrum harmonizing herbs characterized by extensive target promiscuity that could dilute disease-specific pharmacological pathways. Qualifying botanical drugs were advanced to subsequent network target prediction. Active ingredients and targets were retrieved from TCMSP (https://tcmspw.com/) and TCMIP (https://www.tcmip.cn/), filtering for oral bioavailability (OB) ≥ 30% and drug-likeness (DL) ≥ 0.18 (18). Following literature validation, targets were standardized to human gene symbols via UniProt (https://www.uniprot.org/). Cholelithiasis-related disease targets were collected from GeneCards (https://www.genecards.org/), OMIM (https://www.omim.org/), and DrugBank (https://go.drugbank.com/). The intersection of ingredient and disease targets defined the potential therapeutic targets for “component-target” network construction. A protein-protein interaction (PPI) network was built using STRING (https://string-db.org/; confidence ≥ 0.7). Key targets were screened via a two-step strategy: retaining nodes with a degree ≥ twice the median (18) and then ranking the top 10 key targets using the Maximal Clique Centrality (MCC) algorithm in cytoHubba (19). Functional annotation and pathway enrichment analyses were performed using the clusterProfiler package in R (v4.4.3), utilizing the whole-genome annotation database (org.Hs.eg.db) as the statistical background. Significance thresholds were adjusted via the Benjamini–Hochberg method (*p* < 0.05). To ensure methodological transparency and evaluate signal robustness, enrichment was executed in parallel across all 87 overlapping targets (panoramic profile) and the top 10 key targets (modular focus). The identified functional terms and pathways were explicitly defined as exploratory mechanistic hypotheses sensitive to upstream computational filtering criteria.

### 2.10 Molecular docking analysis

To evaluate putative ligand–receptor interactions, molecular docking was conducted for the top three key targets prioritised via topological network analysis alongside their corresponding bioactive constituents. Three-dimensional compound structures were retrieved from TCMSP, energy-minimised, and converted into PDBQT format with assigned Gasteiger charges and flexible rotatable bonds in AutoDockTools (v1.5.6). High-resolution human crystallographic or cryo-electron microscopic (cryo-EM) receptor structures corresponding to the identified hub targets were obtained from the RCSB Protein Data Bank (https://www.rcsb.org/), prioritised by resolution, wild-type sequence completeness, and intact backbone coordinates. Receptors were processed in PyMOL (v3.1.8) by stripping water molecules, solvent ions, and heteroatoms, followed by the addition of polar hydrogens and Kollman charges in AutoDockTools.Global blind docking was executed using AutoDock Vina (v1.1.2) with a grid box configured to envelope the entire macromolecular surface (search exhaustiveness = 8). A binding affinity score ≤ 5 kcal/mol was applied as an exploratory threshold indicating favourable theoretical geometric complementarity. Optimal binding poses exhibiting the lowest docking scores were visualised in PyMOL, and non-covalent intermolecular contacts were mapped via the PLIP platform (https://plip-tool.biotec.tu-dresden.de/). In the absence of active-site re-docking against co-crystallised reference ligands, these docking scores were evaluated strictly as computational indicators of steric and electrostatic compatibility.

## 3 Results

### 3.1 Clinical evidence based on network meta-analysis

#### 3.1.1 Literature screening process and results

A total of 1,201 relevant records were initially identified through database searching, from which 632 records remained after removing duplicates. Following initial screening based on titles and abstracts, alongside a comprehensive full-text review, studies failing to meet the eligibility criteria or containing incomplete data were excluded. Ultimately, 13 RCTs were included for analysis (20–32). One study (33) was excluded for quasi-random allocation based on presentation order, violating the CONSORT requirements for genuine random sequence generation. The detailed screening process is illustrated in Figure 1.

**Figure 1:**
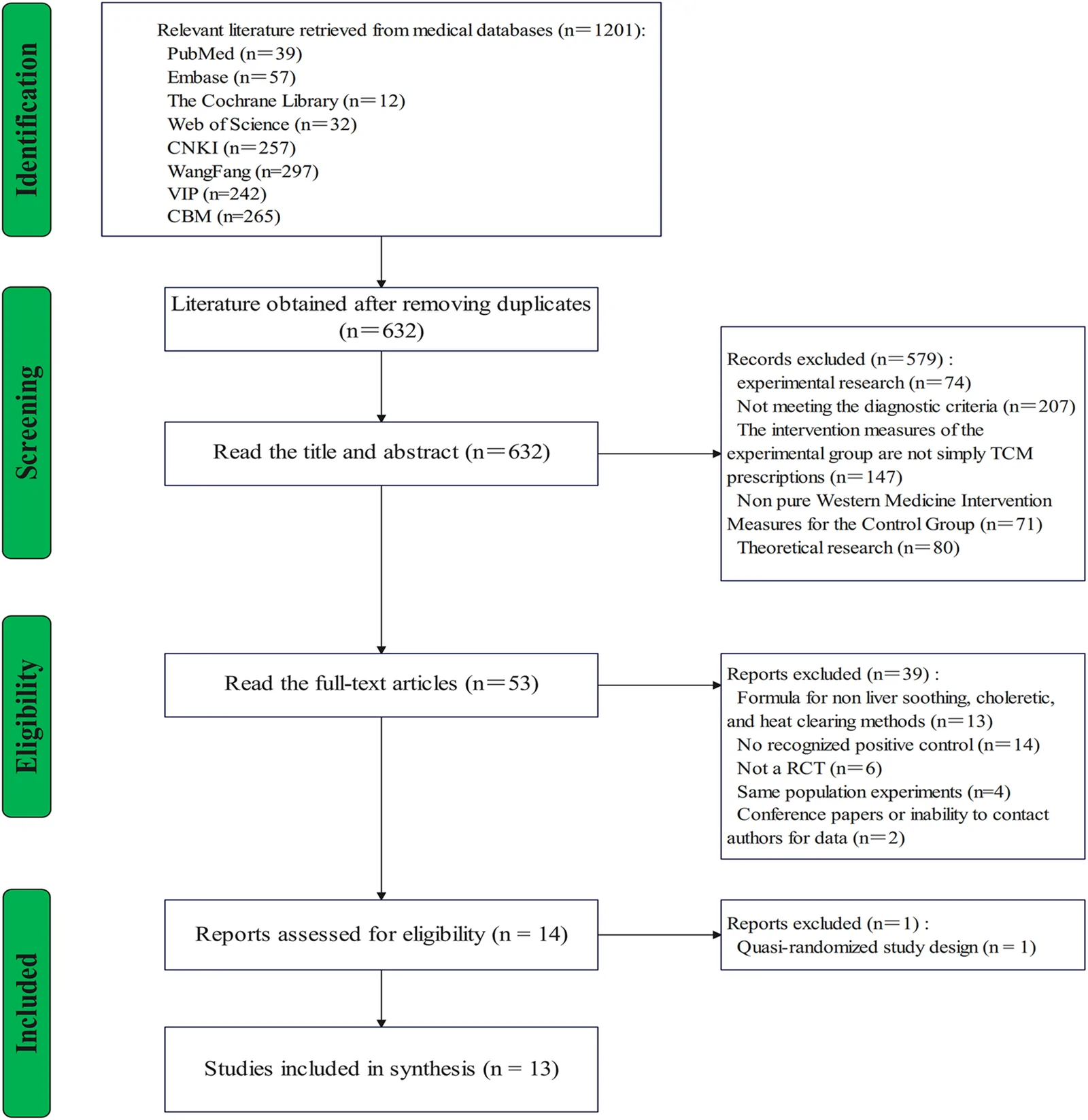
PRISMA flow diagram of study selection and identification.

#### 3.1.2 Basic characteristics of the included studies

This review analyzed 13 Chinese RCTs involving 1,364 patients (694 in the experimental group, 670 in the control group). The included trials were published between 2004 and 2021, with diagnostic definitions primarily informed by authoritative national clinical guidelines or standard monographs (Supplementary Table S1). Specifically, seven studies (21, 24, 25, 27, 29–31) adopted the *Guiding Principles for Clinical Research of New Chinese Medicines (2002)*, of which three (29–31) concurrently referenced the *Consensus on Diagnosis and Treatment of Cholelithiasis with Integrated Traditional Chinese and Western Medicine (2017)* and *Practical Surgery (4th edition)*. Three trials referenced earlier guideline versions or specialised gastroenterology texts (20, 22, 32), whilst the remaining three did not explicitly specify their referenced guideline editions (23, 26, 28). Despite chronological transitions and edition discrepancies in the cited guidelines, all trials rigorously anchored patient baseline eligibility and therapeutic response criteria to objective imaging parameters—specifically, sonographic or radiological assessments of calculus anatomical site, count, and size (Supplementary Tables S2, S3). This objective radiologic verification accords directly with the latest diagnostic criteria outlined in the *Expert Consensus on Integrated Traditional Chinese and Western Medicine Diagnosis and Treatment of Cholelithiasis (2025)* (11), which mandates definitive calculus signs on colour Doppler ultrasound, CT, or MRI. Consequently, this shared imaging baseline guarantees substantial clinical homogeneity and firmly underpins the transitivity assumption across comparison networks. Regarding interventional regimens, experimental groups in all included trials were administered oral TCM decoctions. Across the 13 included studies, the interventions comprised 12 distinct SGLDQR formulas: one trial each evaluated RPDSJN, SNSJJ, JZHF, ZNDST, ZNJJDST, QGLDPSW, ZNLDSJT, SGHST, SJLDT, ZNSGLDT, and SGLDQRF, while two trials evaluated GDSW (standardized full names and herbal compositions of all formulas are detailed in Supplementary Table S4). Control groups were consistently treated with active conventional oral pharmacotherapies (specifically UDCA, CDCA, or ANT; detailed dosing schedules and daily regimens are provided in Supplementary Table S5). Comprehensive baseline patient profiles—including age distribution, disease duration, treatment duration, and reported outcome measures—are summarised in Table 2. The therapeutic course across all eligible trials was at least one month, and baseline demographic and clinical characteristics were well-balanced across treatment arms, demonstrating sound clinical comparability and underpinning the transitivity assumption.

**Table 2:** Basic characteristics of the included studies.

Study	Sample size		Average age		Disease duration		Disease duration		Treatment Duration		Outcome Indicator
	T	C	T	C	T	C	T	C	T	C	
Zhao and Liu, 2004 (20)	100	100	42	45	8	6	RPDSJN	UDCA	6	6	①③④⑤
Xu et al. 2006 (21)	62	64	67.3 ± 16.5	68.5 ± 18.3	-	-	SNSJJ	UDCA	3	3	①
Guo, 2009 (22)	30	30	46.3	45.4	-	-	GDSW	ANT	2	2	①
Xing et al. 2009 (23)	100	101	42	42.03	-	-	JZHF	CDCA	3	3	①⑤
Tan, 2010 (24)	45	36	-	-	1.54	-	ZNDST	UDCA	1	1	①
Shi, 2012 (25)	44	38	40.8 ± 6.2	42.1 ± 6.6	5.8 ± 2.3	6.1 ± 2.8	ZNJJDST	UDCA	6	6	①⑤
Xu and Dang, 2013 (26)	30	30	47.5	46.2	-	-	GDSW	ANT	2	2	①
Dai et al., 2013 (27)	55	45	36.7 ± 13.1	40.3 ± 11.1	1.025 ± 1.133	1.10 ± 0.975	QGLDPSW	UDCA	6	6	①⑤
Guo et al. 2015 (28)	50	50	41.3 ± 2.5	40.7 ± 1.8	1.0 ± 0.6	1.1 ± 0.4	ZNLDSJT	UDCA	3	6	①
Wang, 2019 (29)	50	49	53.74 ± 15.88	51.29 ± 15.91	-	-	SGHST	UDCA	6	6	①②⑤
Zhao, 2020 (30)	49	49	53.29 ± 15.48	50.96 ± 14.78	2.13 ± 0.64	2.17 ± 0.65	SJLDT	UDCA	6	6	①②③④⑤
Li et al. 2021 (31)	40	40	49.1 ± 3.6	51.1 ± 3.3	5.5 ± 1.6	5.3 ± 1.7	ZNSGLDT	UDCA	2	2	①②
Yu, 2021 (32)	39	38	47.67 ± 5.18	48.74 ± 5.06	-	-	SGLDQRF	UDCA	6	6	①②③④⑤

T, experimental groups; C, control groups. Abbreviations for Chinese herbal formulae: RPDSJN, Rongpai Gallstone-Dissolving Capsule; SNSJJ, Modified Sini Powder; XFSHC, Modified Xiaofan Powder; GDSW, Gandan Shu Pill; JZHF, Jinzhi Huang Formula; ZNDST, Self-Designed Gallstone Decoction; ZNJJDST, Self-Designed Jinji Gallstone Decoction; QGLDPSW, Qinggan Lidan Paishi Pill; ZNLDSJT, Self-Designed Lidan Sanjin Decoction; SGHST, Shugan Huashi Decoction; SJLDT, Sijin Lidan Decoction; ZNSGLDT, Self-Designed Shugan Lidan Decoction; SGLDQRF, Shugan Lidan Qingre Formula. UDCA, ursodeoxycholic acid; ANT, anetholtrithione; CDCA, chenodeoxycholic acid. Outcomes assessed: ① clinical efficacy; ② TCM syndrome score; ③ aspartate aminotransferase (AST); ④ alanine aminotransferase (ALT); ⑤ adverse reactions. “-” indicates no data available.

#### 3.1.3 Risk of bias assessment

Methodological quality across the 13 included trials was evaluated using the Cochrane RoB 2.0 (Figure 2). All 13 trials reported randomised allocation: five specified the use of random number tables (24, 26, 30–32), one employed a lottery method (29), and seven omitted precise details of sequence generation. However, because none of the trials provided explicit details on allocation concealment (e.g., sequentially numbered opaque sealed envelopes) and pre-registered trial protocols were universally absent, all studies were conservatively graded as having “Some concerns” in both the “Randomisation process” and “Selection of the reported result”. In “Measurement of the outcome”, the universal absence of double-blinding (open-label design) meant that clinical response rates, TCM syndrome scores, and adverse event reporting were susceptible to subjective reporting and assessor knowledge of assignment, warranting an adjudication of “Some concerns”. “Deviations from intended interventions” and “Missing outcome data” were consistently evaluated as “Low risk” across all studies.

**Figure 2:**
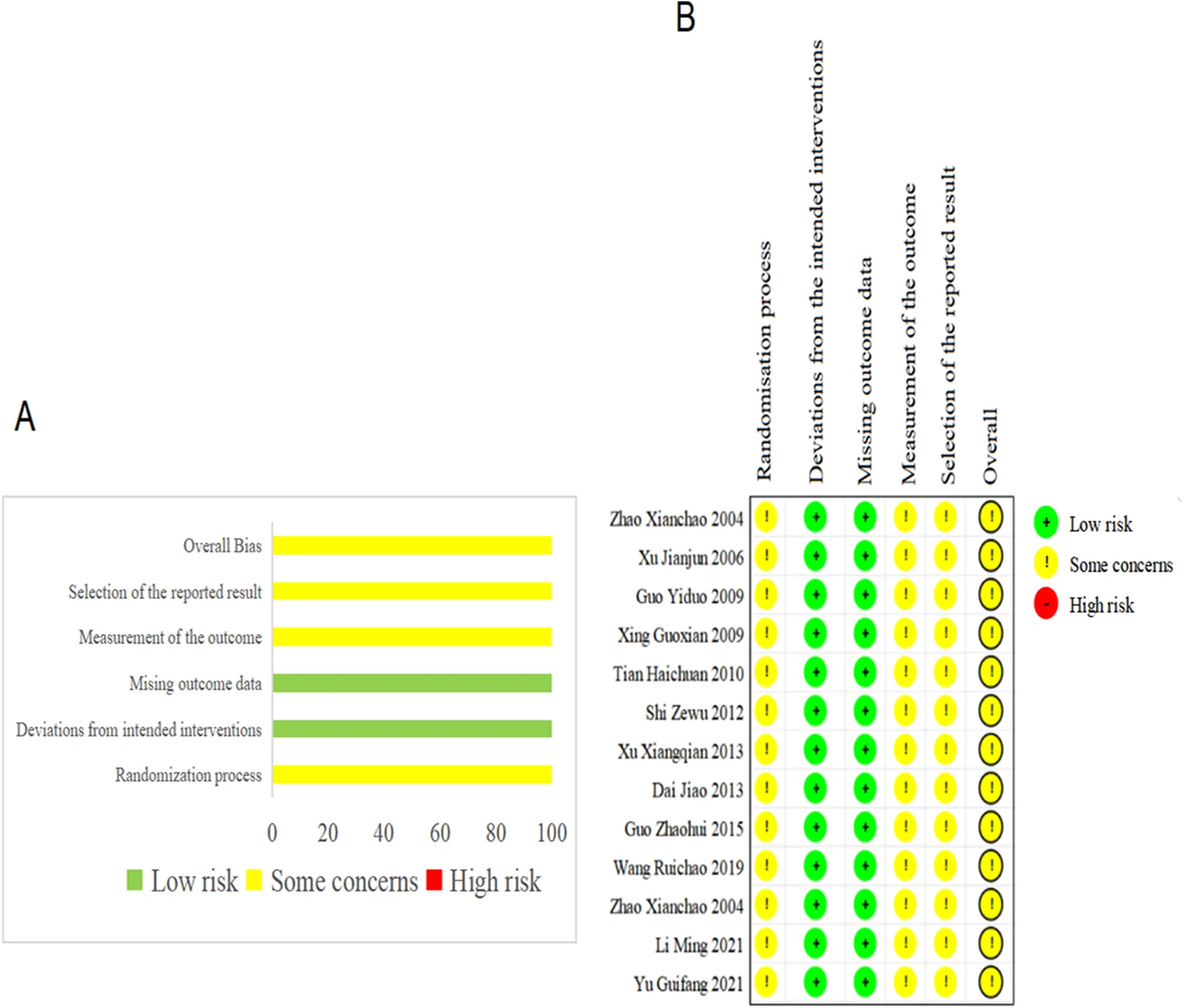
Risk of bias summary **(A)** and bias graph **(B)**.

#### 3.1.4 Evidence network

The evidence network is illustrated in Figure 3, where node sizes and line thicknesses correspond to the sample sizes and numbers of included studies, respectively. Across all evaluated outcomes, each TCM intervention was compared exclusively with WM controls, resulting in a star-shaped network topology that lacked closed loops. Consequently, conventional loop-based local inconsistency testing (e.g., the node-splitting approach) was mathematically infeasible, and the validity of indirect comparisons relied fundamentally on the transitivity assumption. For total clinical efficacy, in addition to the primary analysis against overall conventional WM (Figure 3A), a dedicated sensitivity analysis restricted solely to UDCA controls was performed to verify the robustness of the primary comparator regimen (Figure 3B). Furthermore, for TCM syndrome scores (Figure 3C) and serum AST and ALT levels (Figure 3D), these evidence networks naturally took the form of direct star comparisons between various TCM formulas and UDCA, as these continuous endpoints were reported exclusively by trials that utilized UDCA as the active control.

**Figure 3:**
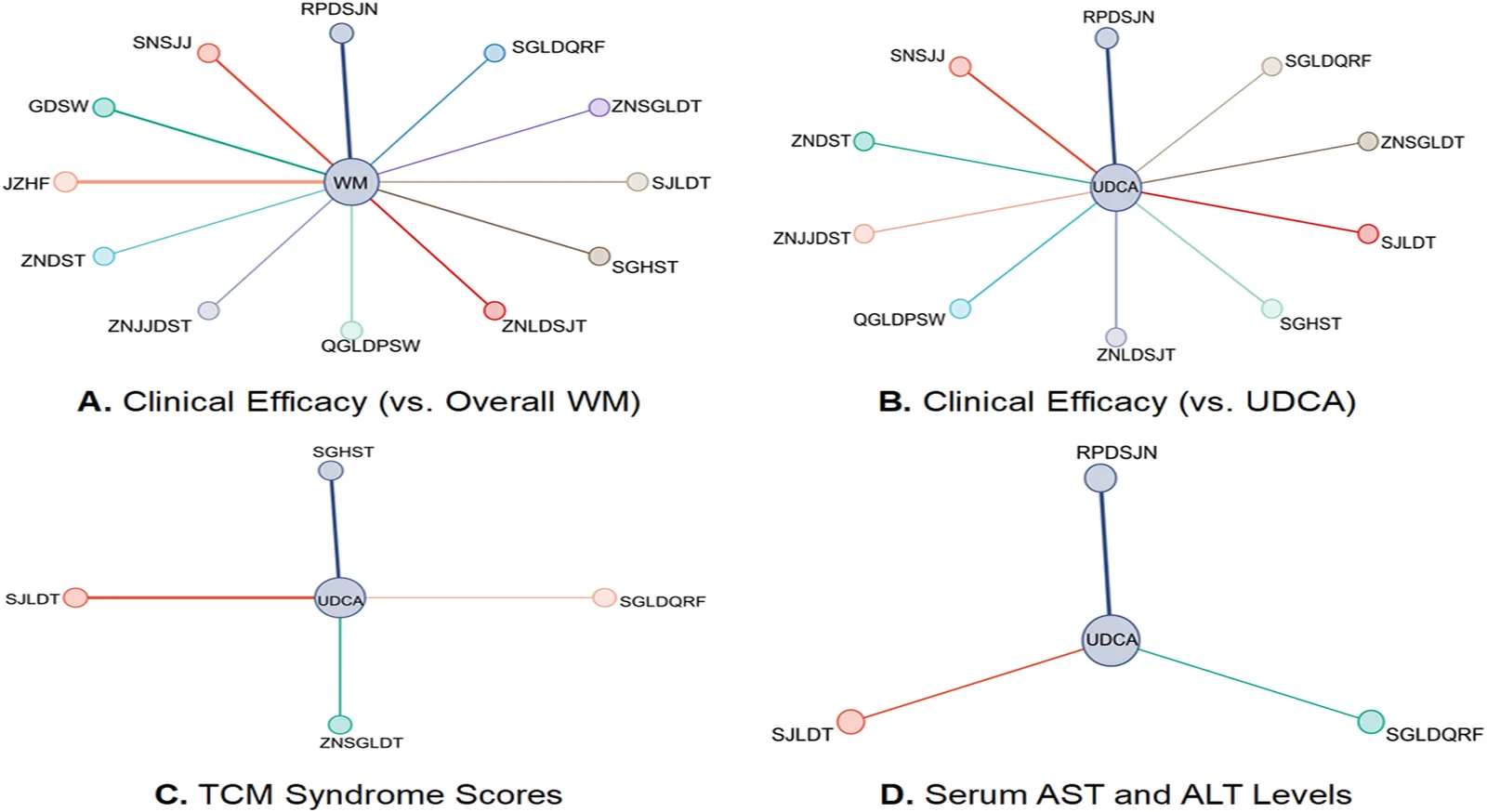
Network meta-analysis evidence diagram. **(A)** Total clinical efficacy versus overall Western medicine (WM) (primary analysis); **(B)** Sensitivity analysis of total clinical efficacy versus ursodeoxycholic acid (UDCA) as the sole comparator; **(C)** TCM syndrome scores (versus UDCA, as reported exclusively by UDCA-controlled trials); **(D)** Serum AST and ALT levels (versus UDCA, as reported exclusively by UDCA-controlled trials). Node sizes correspond to the total number of participants allocated to each intervention. Edge widths are proportional to the number of trials directly comparing the connected nodes.

#### 3.1.5 Clinical efficacy

A total of 13 RCTs were analyzed (20–32). Direct pairwise meta-analysis revealed no statistically significant heterogeneity across studies (*I*^2^ = 0.00%, *p* = 0.585); therefore, a fixed-effects model was applied. The primary analysis demonstrated that SGLDQR TCM formulas achieved significantly superior total clinical efficacy compared with conventional WM [*RR* = 1.52, 95% *CI* (1.42, 1.63)] (Figure 4). To test the robustness of the model specification, a sensitivity analysis using a random-effects model was performed, which yielded virtually identical pooled estimates (*I*^2^ = 0.00%, *p* = 0.623) (Supplementary Figure S1). To evaluate potential effect modifiers and further validate the transitivity assumption required for NMA, predefined subgroup analyses were conducted stratified by types of WM controls (i.e., UDCA, ANT, and CDCA) and treatment durations (1–3 months vs. 4–6 months); clinical efficacy trends remained consistent across all subsets (Supplementary Table S6). Furthermore, univariable meta-regression indicated that neither the type of WM comparator (*t* = −1.52, *p* = 0.157) nor treatment duration (*t* = 0.23, *p* = 0.826) exerted a significant effect modification on clinical efficacy (Supplementary Table S7), thereby confirming the clinical comparability across treatment arms and reinforcing the validity of the transitivity assumption. NMA results revealed that, in the primary analysis comparing against conventional WM as the common control, all evaluated TCM formulas achieved significantly superior total clinical efficacy, with the lower limits of the 95% *CI*s for all *RR*s strictly exceeding 1.00 (the comprehensive pairwise league table is detailed in Supplementary Table S8). The cumulative ranking probabilities assessed by the SUCRA are illustrated in Figure 5A. The ranking results indicated that ZNDST had the highest probability of being the most effective regimen [*RR* = 2.08, 95% *CI* (1.39, 3.13); SUCRA = 88.2%], followed by ZNSGLDT [*RR* = 2.00, 95% *CI* (1.16, 3.45); SUCRA = 81.1%], QGLDPSW [*RR* = 1.72, 95% *CI* (1.20, 2.44); SUCRA = 70.1%], RPDSJN [*RR* = 1.67, 95% *CI* (1.39, 1.96); SUCRA = 69.7%], and ZNJJDST [*RR* = 1.67, 95% *CI* (1.16, 2.44); SUCRA = 67.1%], whereas conventional WM ranked lowest among all evaluated regimens. To further verify the robustness of the primary comparator regimen, a sensitivity analysis was performed by excluding trials with non-UDCA controls (i.e., GDSW controlled with ANT and JZHF controlled with CDCA), thereby retaining exclusively trials utilizing UDCA as the comparator (SUCRA curves are shown in Figure 5B; the corresponding pairwise league table is presented in Supplementary Table S9). Within this UDCA-restricted sub-network, the effect estimates of the TCM formulas remained stable, and the relative hierarchy of the remaining interventions (ZNDST > ZNSGLDT > QGLDPSW > RPDSJN > ZNJJDST > SNSJJ > SGHST > ZNLDSJT > SJLDT > SGLDQRF) was completely identical to that observed in the primary analysis (Supplementary Table S10). This consistency confirms that the variation in Western medicine controls did not substantially bias the relative effects or hierarchical rankings of the interventions, thereby strongly corroborating the robustness of the primary network findings.

**Figure 4:**
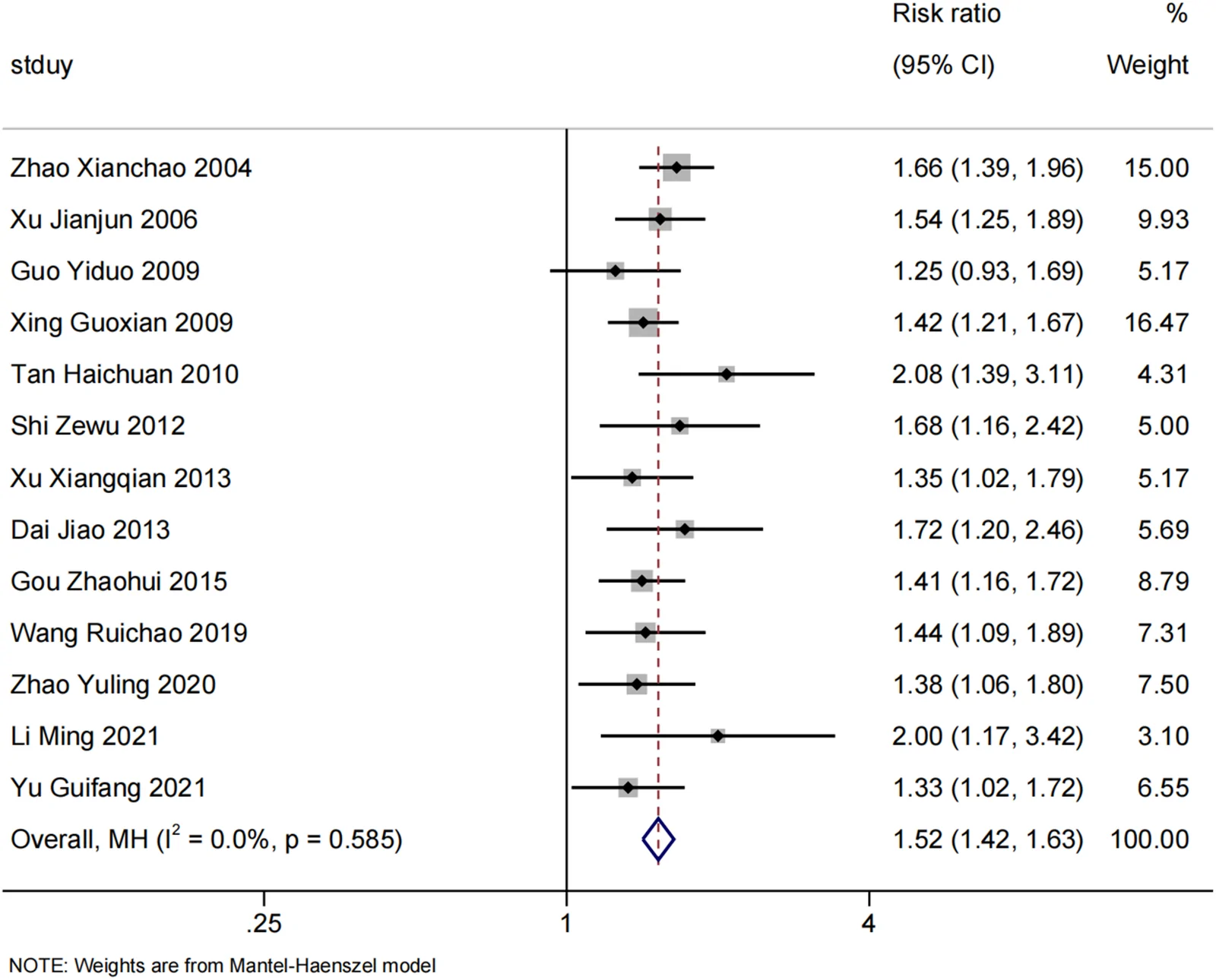
Forest plot of direct pairwise meta-analysis for clinical efficacy using the fixed-effects model.

**Figure 5:**
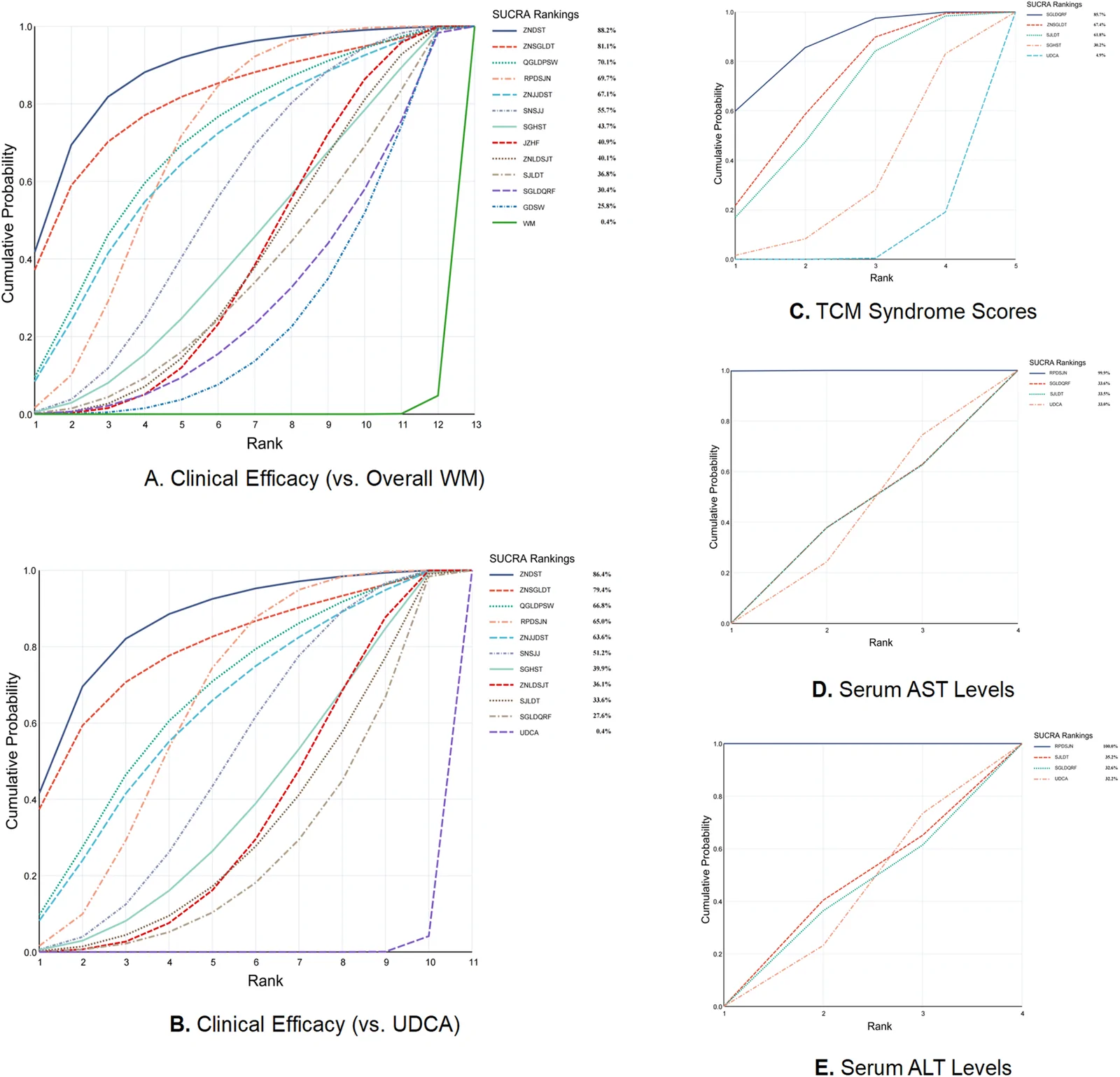
Surface under the cumulative ranking curve (SUCRA) ranking plots for diverse clinical outcomes. **(A)** Clinical efficacy versus overall Western medicine (WM). **(B)** Clinical efficacy versus ursodeoxycholic acid (UDCA). **(C)** TCM syndrome scores. **(D)** Serum AST levels. **(E)** Serum ALT levels.

#### 3.1.6 TCM syndrome scores

Four RCTs (29–32) evaluated TCM syndrome scores (points) using UDCA as the common control. Direct pairwise meta-analysis revealed substantial statistical heterogeneity (*I*² = 87.3%，*p* < 0.001；Cochran's *Q* = 23.68，*df* = 3; Table 3), yielding a pooled *MD* of −3.27 points [95% *CI* (−4.74, −1.80)]. Across the trials, baseline syndrome scores ranged from 11.20 to 12.04 points in the SGHST trial (*MD* = −1.39 points) to 16.75–18.95 points in the remaining three trials (*MD* range: −3.30 to −4.85 points; Supplementary Table S11). Intervention durations were 2 months for ZNSGLDT and 6 months for the other three trials. In the random-effects NMA model, the global between-study variance components were estimated as *τ*² = 1.936 and *τ* = 1.392 (Table 3). Relative to UDCA, significant score reductions were observed for SGLDQRF [*MD* = −4.85 points, 95% *CI* (−7.81, −1.89)], ZNSGLDT [*MD* = −3.62 points, 95% *CI* (−6.47, −0.77)], and SJLDT [*MD* = −3.30 points, 95% *CI* (−6.38, −0.22)], whereas the reduction for SGHST did not reach statistical significance [*MD* = −1.39 points, 95% *CI* (−4.27, 1.49)]. In the pairwise league table (Supplementary Table S12), all comparisons among the four TCM formulas yielded 95% *CI*s spanning the null effect. SUCRA rankings placed the interventions in the following order: SGLDQRF (85.7%), ZNSGLDT (67.4%), SJLDT (61.8%), SGHST (30.2%), and UDCA (4.9%) (Table 4; Figure 5C).

**Table 3:** Heterogeneity analysis results for each outcome.

Outcome	*k*	Q (df)	I²	*τ*²	τ	Random-effects MD [95% CI]
TCM syndrome score	4	23.68 (3)	87.3%	1.936	1.392	−3.27 [-4.74，−1.80]
serum AST levels	3	217.96 (2)	99.1%	525.206	22.917	−32.09 [−58.42，−5.76]
serum ALT levels	3	293.20 (2)	99.3%	482.123	21.957	−41.11 [−66.44，−15.77]

k, number of included studies; Q, Cochran's Q statistic; df, degrees of freedom; I2, percentage of variation attributable to pairwise heterogeneity across studies; tau2 and tau, between-study variance components estimated from the random-effects network meta-analysis model and utilized to construct the pooled confidence intervals; MD, mean difference; CI, confidence interval; TCM, Traditional Chinese Medicine; AST, aspartate aminotransferase; ALT, alanine aminotransferase. Negative MD values indicate greater clinical improvement favoring TCM formulas over the control.

**Table 4:** Random-effects network meta-analysis results for TCM syndrome scores (points).

Intervention	*n* (T/C)	Change from baseline,mean (SD)	UDCA change from baseline,mean (SD)	Random-effects MD [95% CI]	SUCRA
SGHST	50/49	−6.94 (2.18)	−5.55 (2.49)	−1.39 [−4.27，1.49]	30.2%
SJLDT	49/49	−11.10 (3.46)	−7.80 (3.72)	−3.30 [−6.38，−0.22]	61.8%
ZNSGLDT	40/40	−11.57 (2.26)	−7.95 (1.40)	−3.62 [−6.47，−0.77]	67.4%
SGLDQRF	39/38	−12.77 (2.43)	−7.92 (2.70)	−4.85 [−7.81，−1.89]	85.7%

n (T/C), sample size in treatment group/control group; SD, standard deviation; MD, mean difference; CI, confidence interval; SUCRA, surface under the cumulative ranking curve; UDCA, ursodeoxycholic acid; TCM, Traditional Chinese Medicine.

#### 3.1.7 Serum AST and ALT levels

Three RCTs (20, 30, 31), each evaluating a 6-month treatment course, reported serum AST and ALT levels against UDCA. Direct pairwise meta-analyses demonstrated substantial statistical heterogeneity (*I*² = 99.1% for AST; *I*² = 99.3% for ALT; Table 3). Under the random-effects NMA framework, the global between-study variance components were estimated as *τ*² = 525.206 (*τ* = 22.917) for AST and *τ*² = 482.123 (*τ* = 21.957) for ALT. Baseline enzymatic profiles diverged markedly across studies (Supplementary Tables S13, S14). Specifically, the RPDSJN trial enrolled patients with baseline values exceeding physiological upper limits of normal (mean AST: ∼150.00 U/L; ALT: ∼170.00 U/L) (34), achieving notable reductions relative to UDCA [*MD*: AST = −101.00 U/L, 95% *CI* [−147.82, −54.18]; ALT = −131.00 U/L, 95% *CI* [−176.54, −85.46]; Tables 5, 6]. In the pairwise league tables (Supplementary Tables S15, S16), RPDSJN also yielded statistically significant reductions over SGLDQRF [*MD*: AST = −100.90 U/L, 95% *CI* [−165.83, −35.97]; ALT = −131.20 U/L, 95% *CI* [−193.89, −68.51]] and SJLDT [*MD*: AST = −100.66 U/L, 95% *CI* [−165.65, −35.67]; ALT = −129.16 U/L, 95% *CI* [−191.89, −66.43]]. Conversely, patients in the SJLDT and SGLDQRF trials presented with normal baseline transaminases (25–28 U/L; Supplementary Tables S13, S14). Consequently, all head-to-head comparisons among SJLDT, SGLDQRF, and UDCA yielded near-zero effect sizes with wide 95% CIs spanning the line of no effect. SUCRA rankings placed RPDSJN first for both AST (99.9%) and ALT (100.0%) reductions (Figures 5D,E).

**Table 5:** Random-effects network meta-analysis results for serum AST levels (U/L).

Intervention	*n* (T/C)	Change from baseline,mean (SD)	UDCA change from baseline,mean (SD)	Random-effects MD [95% CI]	SUCRA
RPDSJN	100/100	−99.00 (41.16)	2.00 (53.44)	−101.00 [−147.82，−54.18]	99.9%
SJLDT	49/49	−2.41 (9.46)	−2.07 (9.40)	−0.34 [−45.41，44.73]	33.5%
SGLDQRF	39/38	−1.70 (5.63)	−1.60 (5.70)	−0.10 [−45.09，44.89]	33.6%

AST, aspartate aminotransferase; T/C, treatment/control; SD, standard deviation; UDCA, ursodeoxycholic acid; MD, mean difference; CI, confidence interval; SUCRA, surface under the cumulative ranking curve.

**Table 6:** Random-effects network meta-analysis results for serum ALT levels (U/L).

Intervention	*n* (T/C)	Change from baseline,mean (SD)	UDCA change from baseline,mean (SD)	Random-effects MD [95% CI]	SUCRA
RPDSJN	100/100	−128.00 (50.44)	3.00 (56.77)	−131.00 [−176.54，−85.46]	100.0%
SJLDT	49/49	−3.11 (8.01)	−1.27 (7.37)	−1.84 [−44.98，41.30]	35.2%
SGLDQRF	39/38	−1.00 (5.07)	−1.20 (3.84)	0.20 [−42.88，43.28]	32.6%

ALT, alanine aminotransferase; T/C, treatment/control; SD, standard deviation; UDCA, ursodeoxycholic acid; MD, mean difference; CI, confidence interval; SUCRA, surface under the cumulative ranking curve.

#### 3.1.8 Adverse events

Eight studies reported adverse events (20, 22, 25, 27, 29–31). Due to inconsistent recording criteria, only a descriptive analysis was performed. Four trials observed no adverse events or clinical abnormalities (22, 25, 27, 31). The remaining three reported mild events: Zhao & Liu (2004) (20) noted diarrhea (TCM: 11 cases, WM: 67 cases); Wang (2019) (29) reported 3 diarrhea cases in the WM group and Zhao (2020) (30) recorded 1 diarrhea case (group unstated). All events were mild and resolved spontaneously.

#### 3.1.9 Publication bias and sensitivity analysis

For the clinical overall response rate, the comparison-adjusted funnel plot (Figure 6) displayed visual asymmetry. Egger's linear regression test revealed a statistically significant intercept of 0.733 (*p* = 0.033; Figure 7), indicating the presence of small-study effects. In the leave-one-out sensitivity analysis (Supplementary Figure S2), sequential exclusion of individual studies yielded no directional reversals or substantial deviations in the pooled *RR*s and corresponding 95% *CI*s.

**Figure 6:**
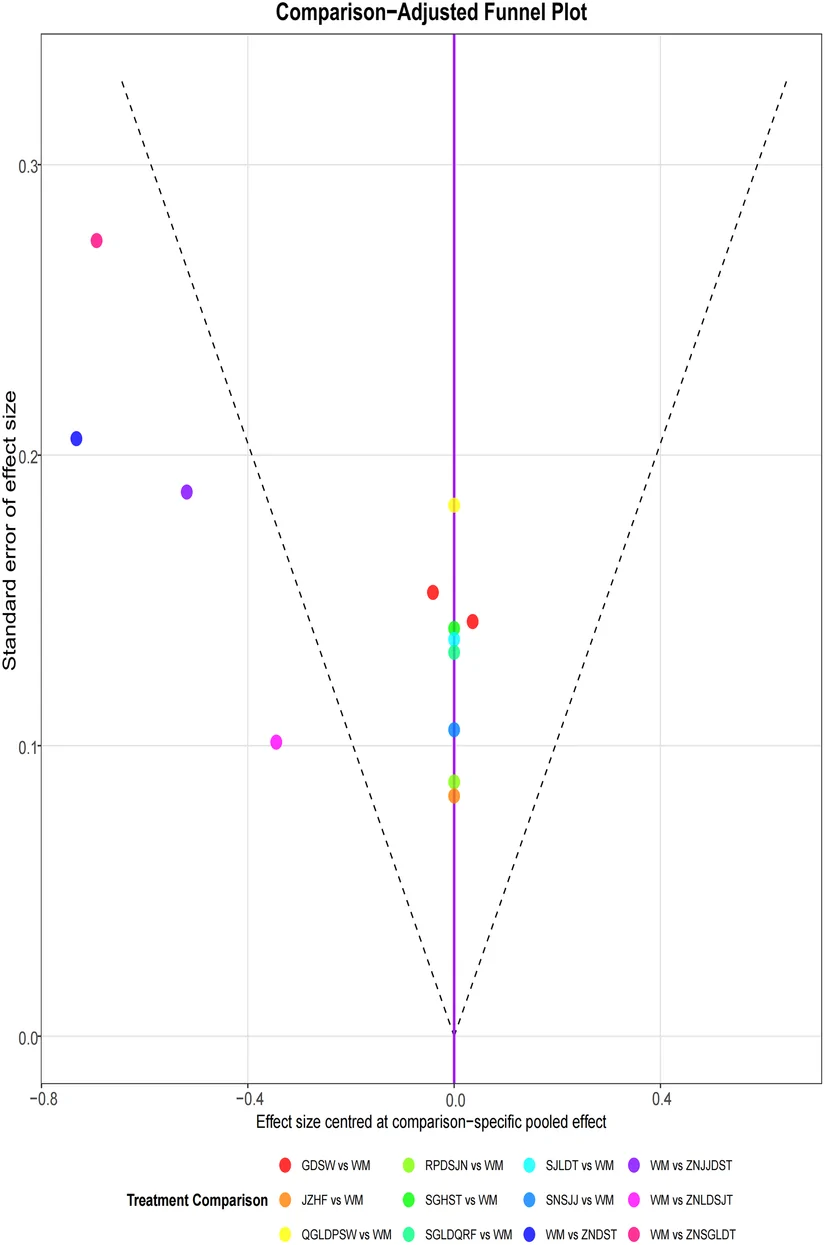
Comparison-adjusted funnel plot for clinical efficacy.

**Figure 7:**
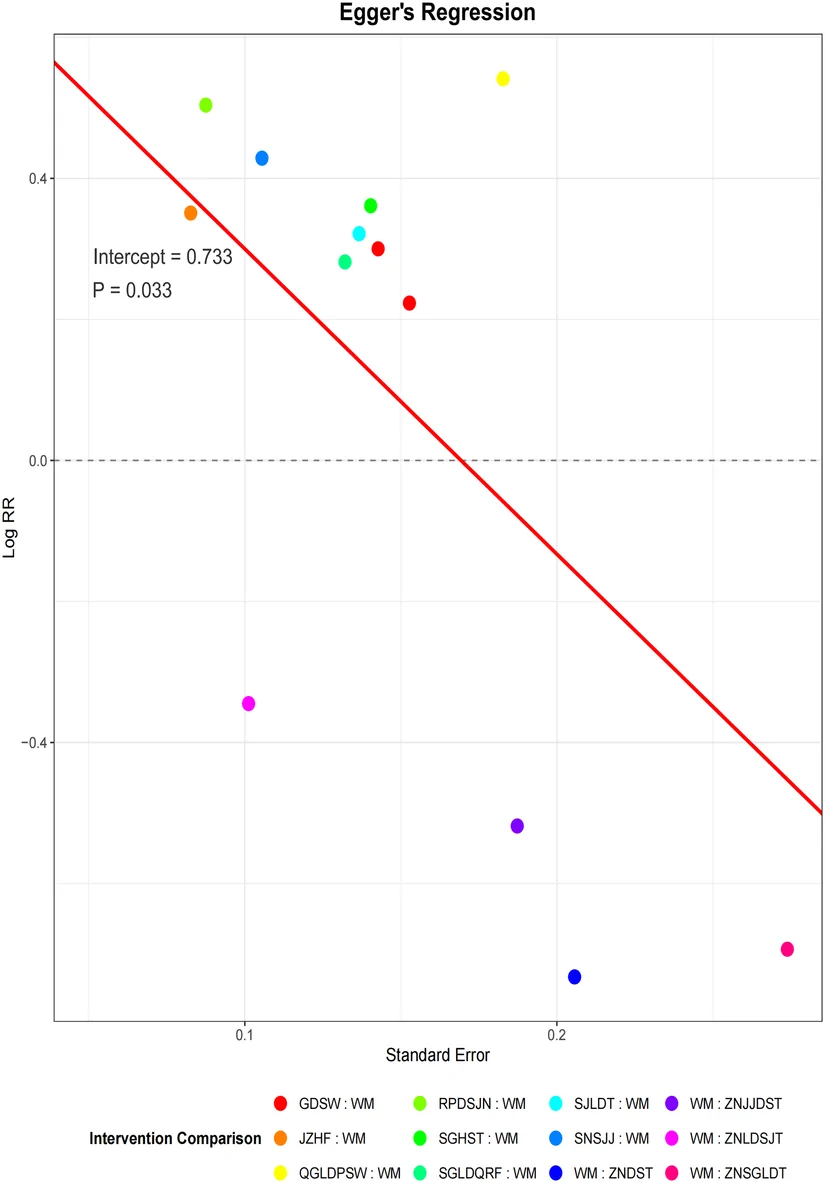
Egger's regression test for clinical efficacy.

#### 3.1.10 Certainty of evidence

According to the CINeMA framework, the confidence ratings for the clinical overall response rate were “low” across all comparisons between the 12 interventions and WM (Table 7). For pairwise comparisons among the active interventions, the certainty of evidence was evaluated as either “low” or “very low” (Supplementary Table S17).

**Table 7:** CINeMA evaluation of evidence certainty for clinical efficacy: 12 interventions vs. WM.

Comparison	Within-study bias	Reporting bias	Indirectness	Imprecision	Heterogeneity	Incoherence	Confidence rating
GDSW:WM	Some concerns	Some concerns	No concerns	No concerns	No concerns	No concerns	Low
JZHF:WM	Some concerns	Some concerns	No concerns	No concerns	No concerns	No concerns	Low
QGLDPSW:WM	Some concerns	Some concerns	No concerns	No concerns	No concerns	No concerns	Low
RPDSJN:WM	Some concerns	Some concerns	No concerns	No concerns	No concerns	No concerns	Low
SGHST:WM	Some concerns	Some concerns	No concerns	No concerns	No concerns	No concerns	Low
SGLDQRF:WM	Some concerns	Some concerns	No concerns	No concerns	No concerns	No concerns	Low
SJLDT:WM	Some concerns	Some concerns	No concerns	No concerns	No concerns	No concerns	Low
SNSJJ:WM	Some concerns	Some concerns	No concerns	No concerns	No concerns	No concerns	Low
ZNDST:WM	Some concerns	Some concerns	No concerns	No concerns	No concerns	No concerns	Low
ZNJJDST:WM	Some concerns	Some concerns	No concerns	No concerns	No concerns	No concerns	Low
ZNLDSJT:WM	Some concerns	Some concerns	No concerns	No concerns	No concerns	No concerns	Low
ZNSGLDT:WM	Some concerns	Some concerns	No concerns	No concerns	No concerns	No concerns	Low

Yellow, some concerns; Green, no concerns; Red, major concerns; Gold, low; Pink, very low.

### 3.2 Data mining-based analysis of TCM prescription rules

#### 3.2.1 Herbal frequency analysis

The compositional formulations of the included prescriptions across the 13 studies are detailed in Supplementary Table S4. Frequency analysis identified a total of 47 distinct botanical drugs (Supplementary Table S18), among which the top 17 high-frequency herbs are illustrated in Figure 8.

**Figure 8:**
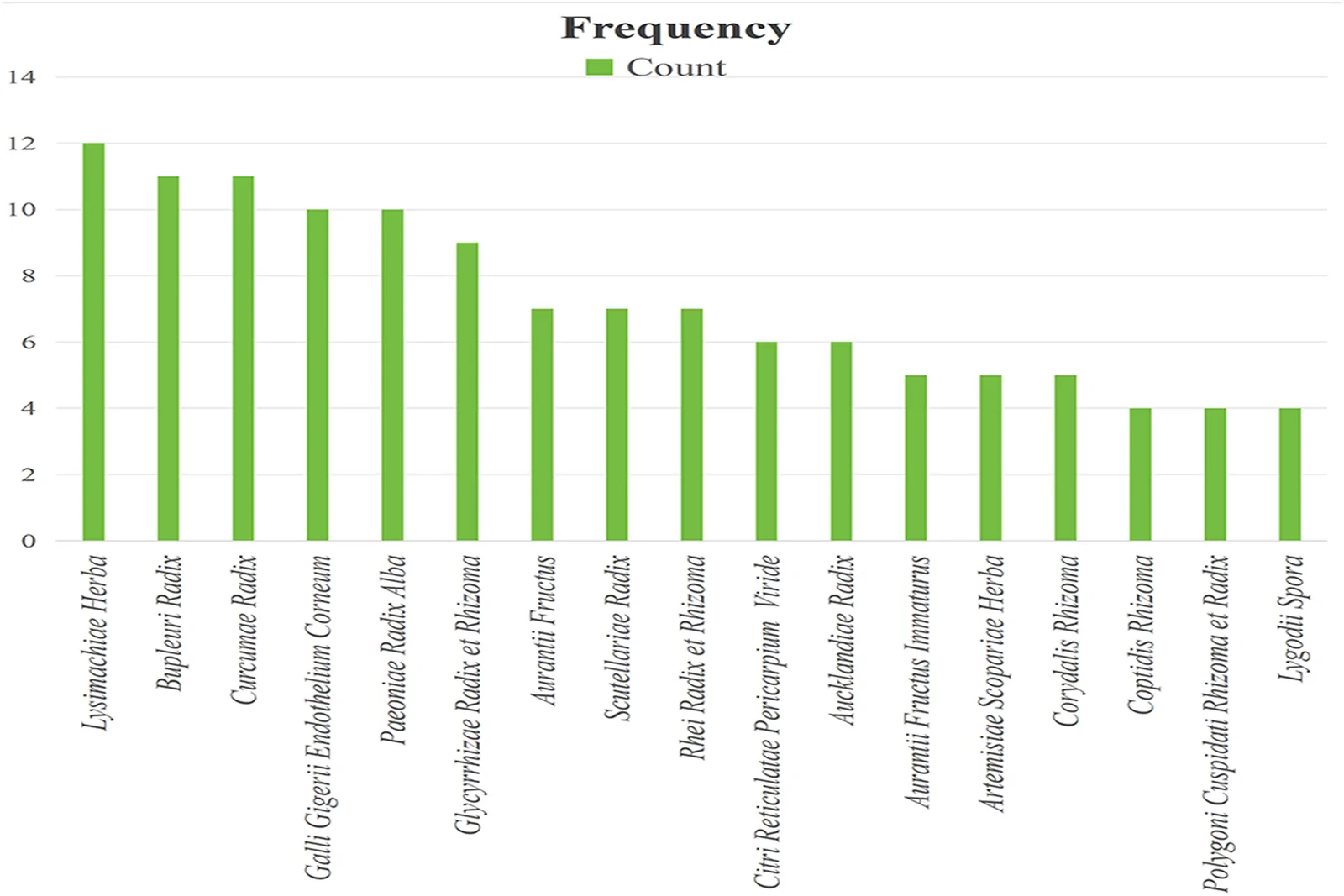
Top 17 herbs by frequency.

#### 3.2.2 Property distributions of included herbs

The properties of the included herbs were analyzed across four dimensions (Figure 9). The top three predominant profiles for each dimension were: slightly cold, cold, and warm for four properties; bitter, pungent, and sweet for five tastes; spleen, liver, and stomach meridians for channel tropisms; and bile-flow-promoting, heat-clearing, and liver-soothing medicinals for functional categories.

**Figure 9:**
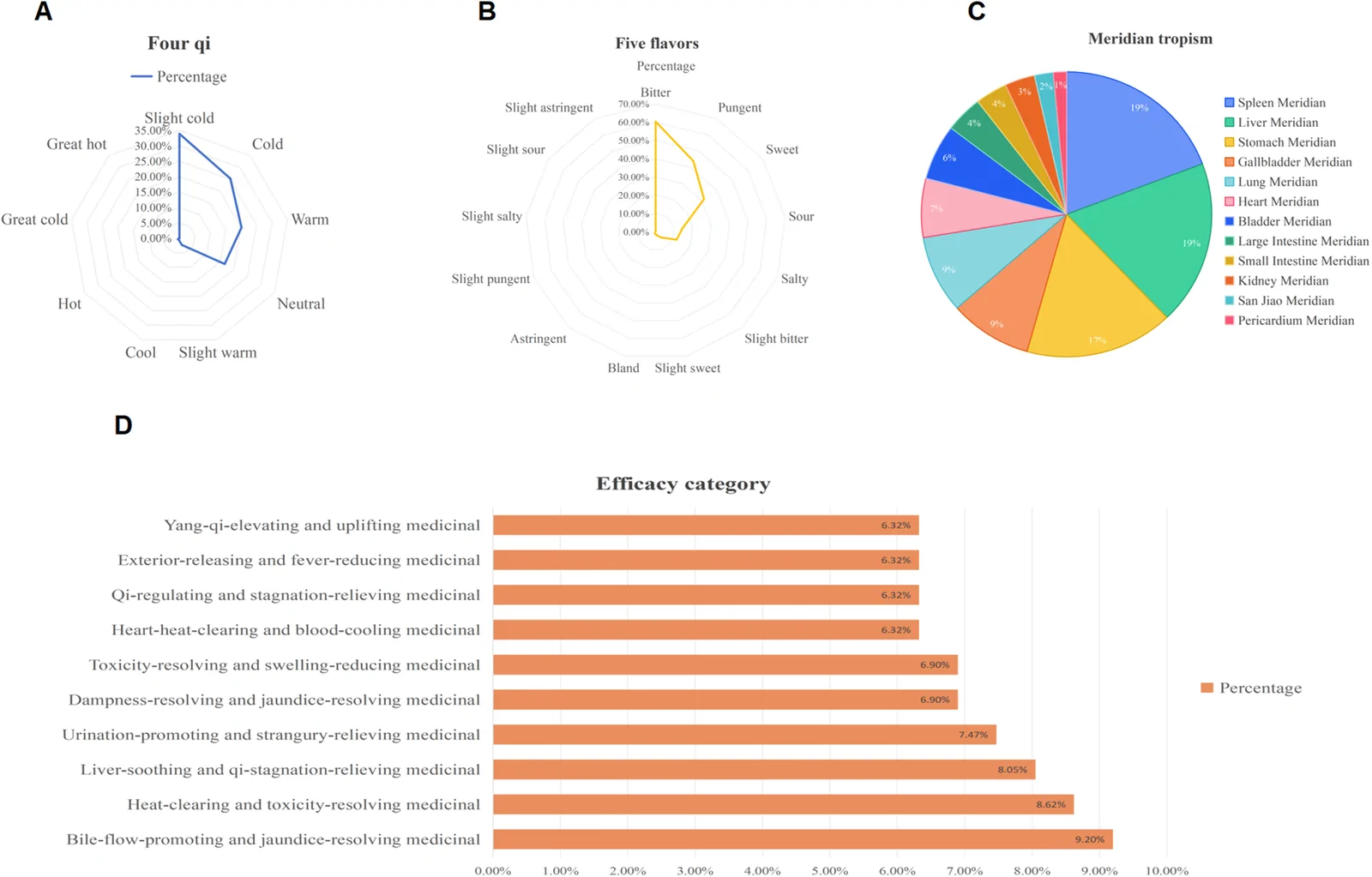
Distribution of herbal properties. **(A)** Four qi. **(B)** Meridian tropism. **(C)** Five flavors. **(D)** Efficacy categories.

#### 3.2.3 Association rule analysis

A total of 25 high-frequency herb pairs were identified based on a minimum support threshold of 50% and a confidence threshold of 80% (Supplementary Table S19). Among these, eight core herbs exhibited the highest association strength: *Gigeriae Galli Endothelium Corneum* (Jineijin)*, Lysimachiae Herba* (Jinqiancao)*, Bupleuri Radix* (Chaihu)*, Curcumae Radix* (Yujin)*, Paeoniae Radix Alba* (Baishao)*, Glycyrrhizae Radix et Rhizoma* (Gancao)*, Rhei Radix et Rhizoma* (Dahuang)*, and Scutellariae Radix* (Huangqin). The interaction network of these core herbs is illustrated in Figure 10.

**Figure 10:**
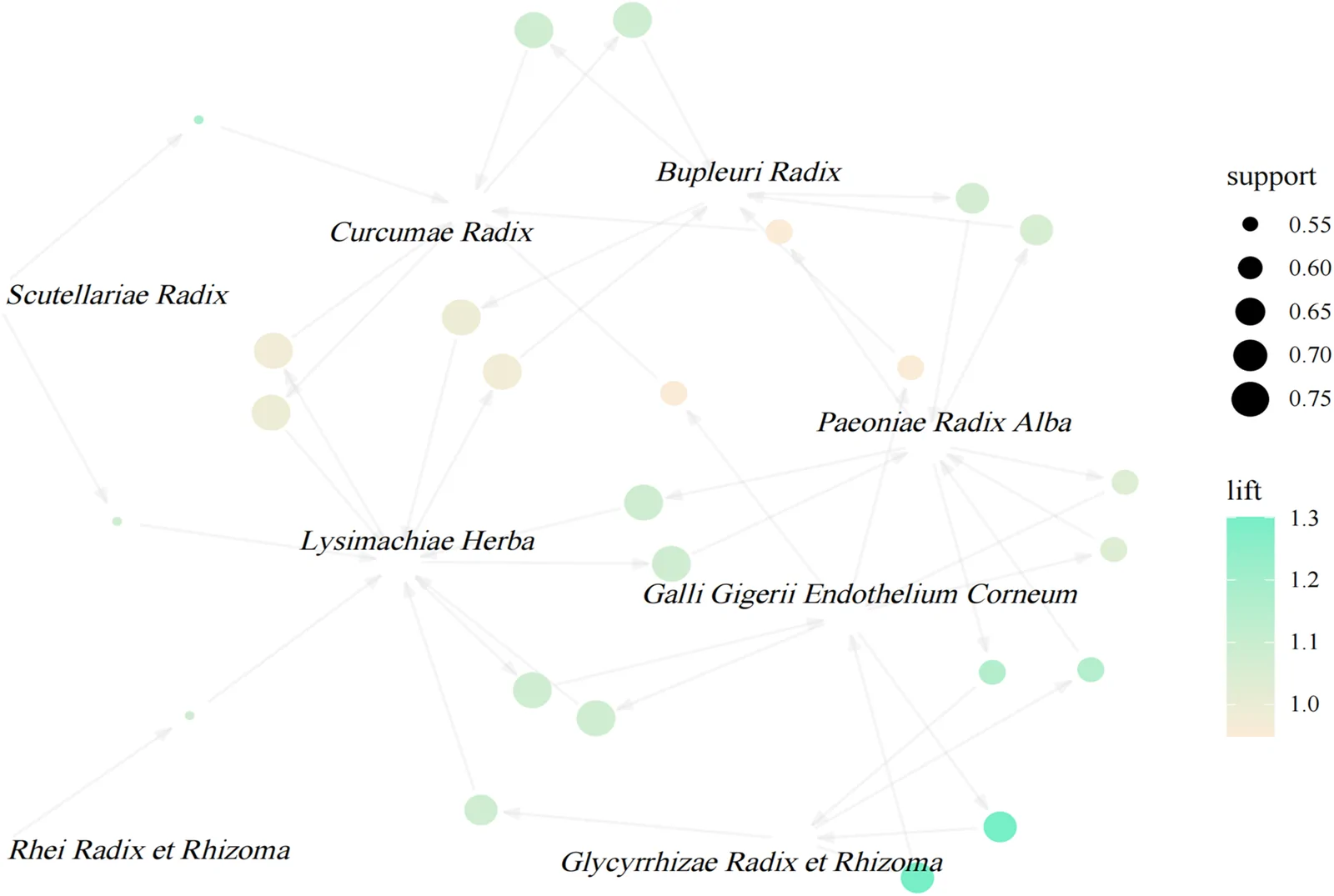
Network diagram of association rules.

### 3.3 Network pharmacology-based mechanism prediction

Combination of frequency analysis and association rule mining initially identified eight candidate botanical drugs. In accordance with the pre-specified criteria for computational feasibility and signal resolution, two agents were excluded prior to target prediction: *Galli Gigerii Endothelium Corneum* (due to insufficient small-molecule compound–target annotations) and *Glycyrrhizae Radix et Rhizoma* (due to extensive target promiscuity as a broad-spectrum harmonizing herb). Consequently, six core botanical drugs—*Lysimachiae Herba* (JQC), *Bupleuri Radix* (CH), *Curcumae Radix* (YJ), *Paeoniae Radix Alba* (BS), *Rhei Radix et Rhizoma* (DH), and *Scutellariae Radix* (HQ)—were retained for downstream target prediction and pathway enrichment analyses.

#### 3.3.1 Identification of key targets for cholelithiasis

An “herb-ingredient-target” network (198 ingredients, 398 targets) (Supplementary Table S20) yielded 87 overlapping targets with cholelithiasis (Figure 11A,B). PPI network analysis via Cytoscape initially screened 23 highly connected targets using cytoNCA (Degree > 32) (Figure 11C). Subsequent ranking via the CytoHubba MCC algorithm identified the top 10 key targets: *IL6, IL1B, TNF, IL10, IFNG, EGFR, CCL2, TLR4, ALB,* and *CXCL8* (Figure 11D, Table 8).

**Figure 11:**
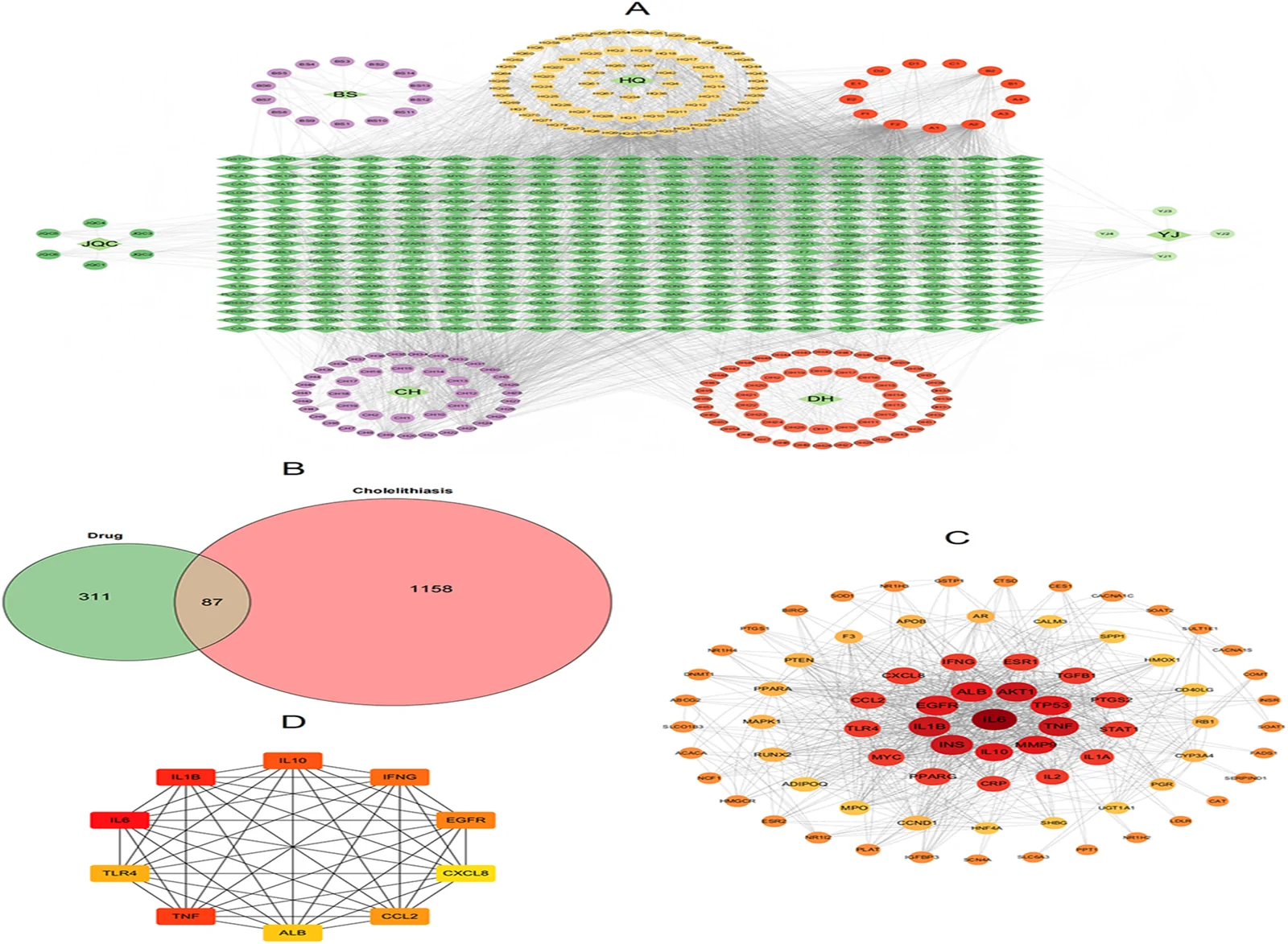
Screening and identification of key targets for cholelithiasis treatment. **(A)** Herb-component-target network. **(B)** Venn diagram of the intersection between herb-derived and disease-related targets. **(C)** Target network ranked by degree. **(D)** Targets ranked by MCC score.

**Table 8:** The most critical ten genes and MMC values.

RANK	1	2	3	4	5	6	7	8	9	10
Gene	*IL6*	*IL1B*	*TNF*	*IL10*	*IFNG*	*EGFR*	*CCL2*	*TLR4*	*ALB*	*CXCL8*
MCC (E9)	3.1648	3.1647	3.1645	3.1630	3.1469	3.1461	3.0596	3.0522	3.0245	2.6168

#### 3.3.2 GO and KEGG enrichment analyses

Functional enrichment was conducted for both the 87 overlapping targets (Supplementary Figures S3, S4) and the top 10 key targets (Figures 12, 13) against the genome-wide background. In the GO analysis, shared biological processes (BP) between both datasets primarily comprised response to lipopolysaccharide, regulation of inflammatory response, cholesterol biosynthetic process, and regulation of lipid metabolic process; shared molecular functions (MF) mapped to cytokine activity. These overlapping functional terms consistently corresponded to immune-inflammatory responses and lipid metabolic processes. In the KEGG analysis, prominent pathways co-enriched among the top-ranked entries included Lipid and atherosclerosis, alongside primary inflammatory cascades such as the IL-17 signaling pathway, cytokine–cytokine receptor interaction, and the AGE-RAGE signaling pathway. These co-identified pathways showed common convergence onto immune-inflammatory and lipid metabolism signaling. Detailed enrichment profiles for the 10 key targets and 87 overlapping targets are presented in Figures 12, 13 and Supplementary Figures S3, S4, respectively.

**Figure 12:**
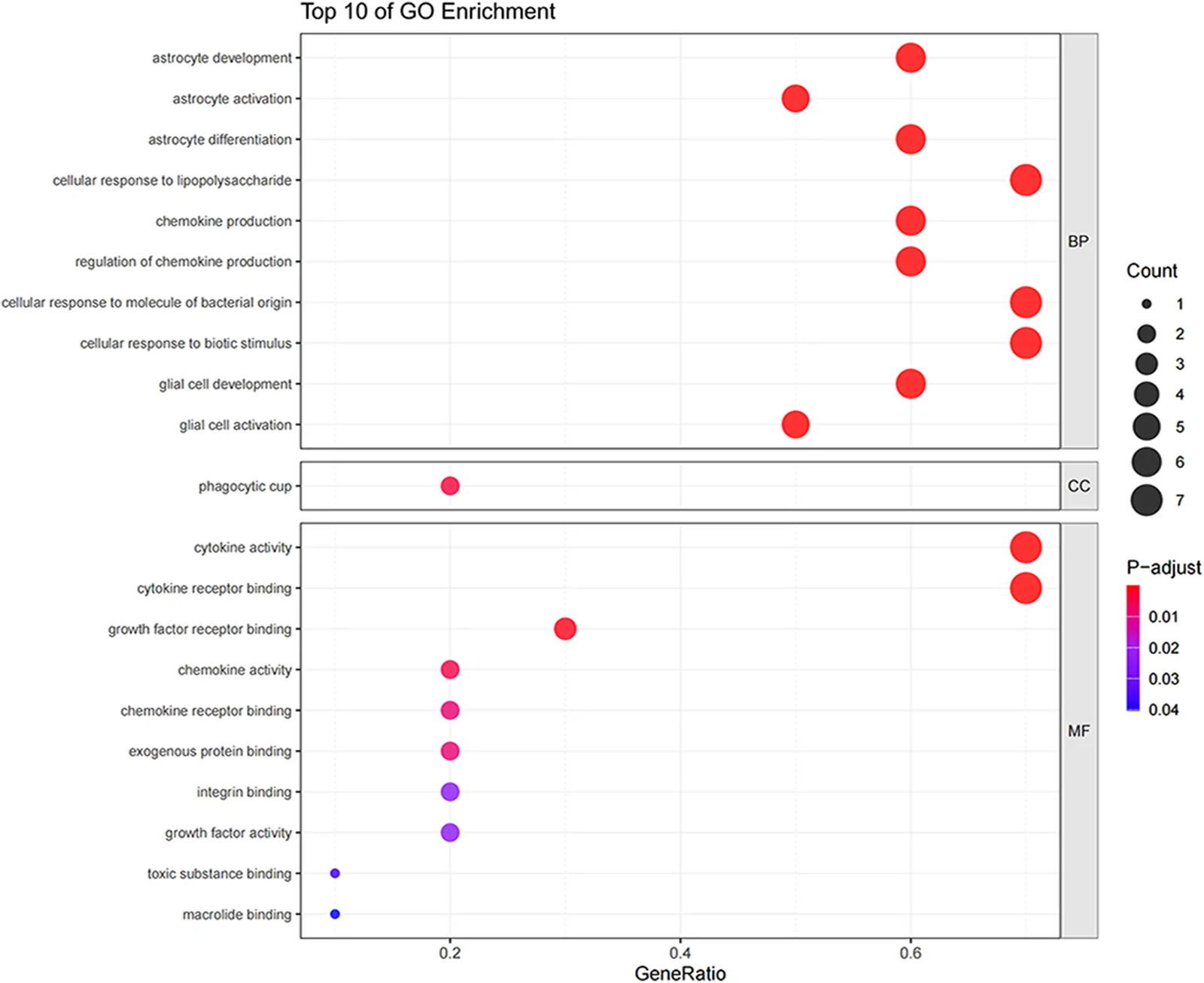
Top 10 gene ontology (GO) enrichment terms for the 10 core targets.

**Figure 13:**
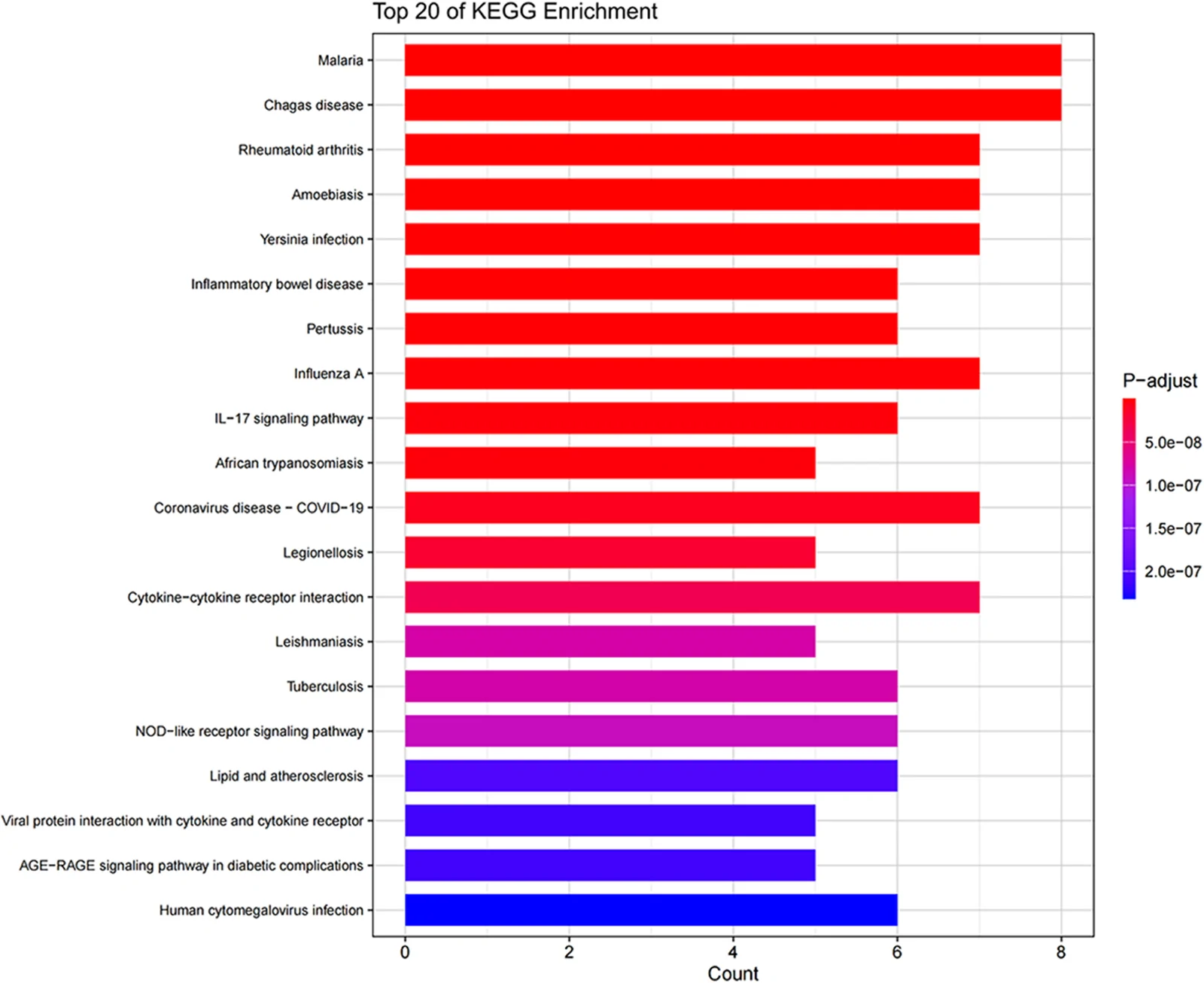
Top 20 Kyoto encyclopedia of genes and genomes (KEGG) pathways enriched by the 10 core targets.

### 3.4 Molecular docking validation based on key targets

Molecular docking was performed between the top three topological key targets (IL-6, IL-1β, and TNF-α) and their corresponding bioactive ingredients. Target gene nomenclature and protein details are provided in Supplementary Table S21, and receptor structural parameters alongside docking grid configurations are summarized in Supplementary Table S22. All docking conformations yielded binding affinities ranging from −6.1 to −7.2 kcal/mol, below the pre-specified threshold of −5.0 kcal/mol (Table 9). The local three-dimensional binding patterns for each complex are shown in Figure 14. In the IL-6/gp130 signaling complex (PDB ID: 8QY6, Chain D), paeoniflorin yielded a binding affinity of −7.1 kcal/mol, forming hydrogen bonds with residues including Thr280 (2.8 Å) and Lys252 (3.3 Å); quercetin (−6.8 kcal/mol), oroxylin A (−6.7 kcal/mol), and wogonin (−6.1 kcal/mol) formed hydrogen bonds with residues including Val465 (2.9 Å), Trp455 (2.9 Å), Asp253 (2.9 Å), and Gln470 (3.0 Å), among others (Figure 14A). In the IL-1β signaling complex (PDB ID: 4DEP, Chain B), quercetin exhibited a binding affinity of −7.2 kcal/mol, forming hydrogen bonds with residues including Lys114 (3.0 Å); aloe-emodin (−6.7 kcal/mol) formed hydrogen bonds with residues including Ile13 (2.9 Å) and Leu15 (3.1 Å), among others (Figure 14B). In the TNFR1-selective TNF mutant (PDB ID: 2ZJC, Chain B), quercetin (−6.8 kcal/mol), wogonin (−6.6 kcal/mol), paeoniflorin (−6.5 kcal/mol), and aloe-emodin (−6.5 kcal/mol) formed hydrogen bonds with residues including Tyr115 (2.8 Å), Gln102 (3.0 Å), Arg103 (3.0 Å), and Ser65 (3.2 Å), among others (Figure 14C).

**Table 9:** Molecular docking scores of core bioactive compounds with primary key targets.

Molecule ID	Molecule Name	Binding Affinity(kcal/mol)		
		IL-6	IL-1β	TNF-α
MOL000098	quercetin	−6.8	−7.2	−6.8
MOL001924	paeoniflorin	−7.1	-	−6.5
MOL000173	wogonin	−6.1	-	−6.6
MOL002928	oroxylin a	−6.7	-	-
MOL000471	aloe-emodin	-	−6.7	−6.5

Molecule identifiers were obtained from the Traditional Chinese Medicine Systems Pharmacology (TCMSP) database. A calculated binding affinity < −5.0 kcal/mol represents the threshold for favorable steric and electrostatic complementarity. “-” indicate that the compound–target pair was not predicted in the upstream network pharmacology screening and was consequently not subjected to docking analysis.

**Figure 14:**
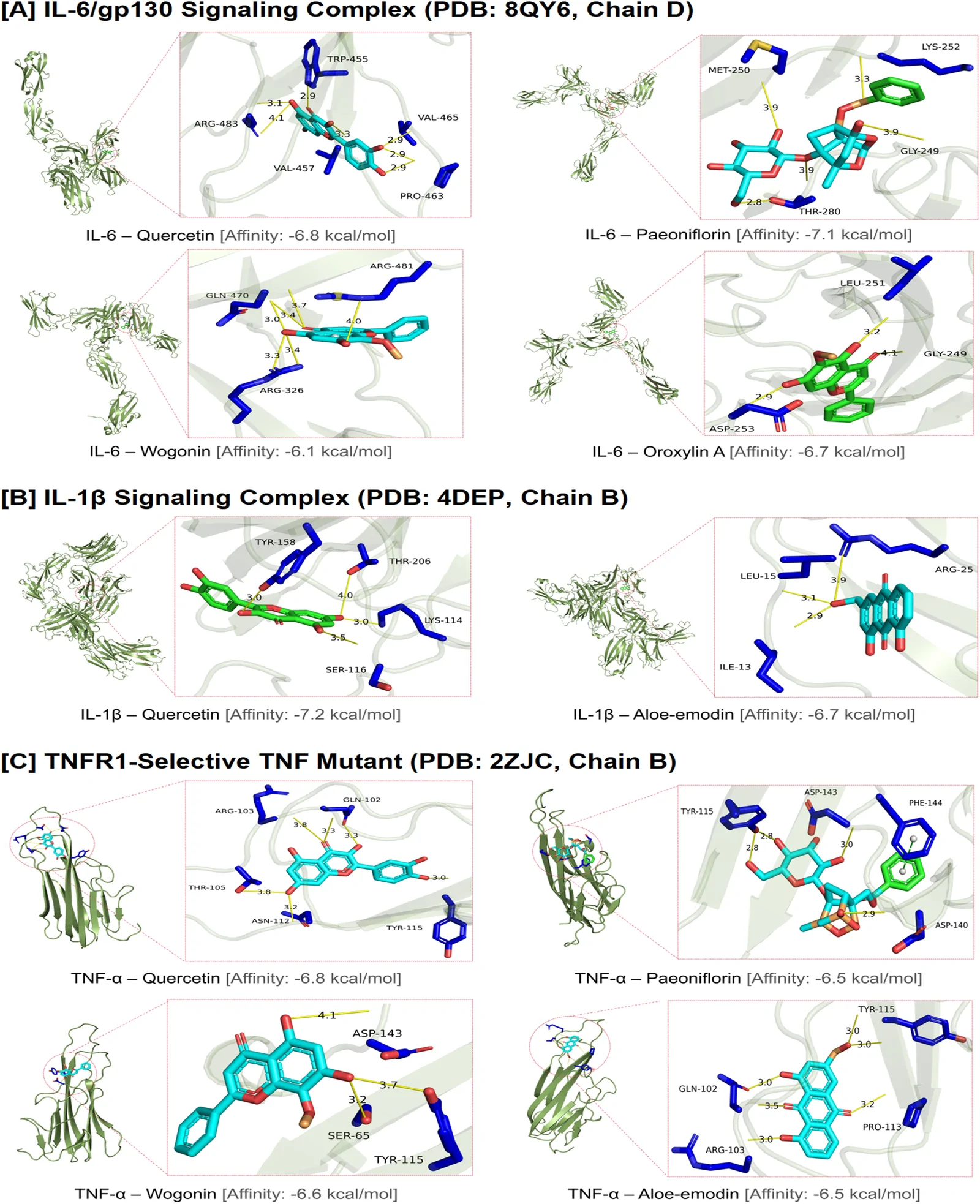
Molecular docking patterns of core active compounds with central inflammatory targets. **(A)** IL-6/gp130 signaling complex (PDB ID: 8QY6, Chain D) complexed with quercetin, paeoniflorin, wogonin, and oroxylin A. **(B)** IL-1β signaling complex (PDB ID: 4DEP, Chain B) complexed with quercetin and aloe-emodin. **(C)** TNFR1-selective TNF mutant (PDB ID: 2ZJC, Chain B) complexed with quercetin, paeoniflorin, wogonin, and aloe-emodin. For each docking profile, the left panoramic view illustrates the global receptor–ligand configuration, in which the target protein chain is depicted in cyan, and the binding pocket is delineated by a red dashed circle. The right close-up view displays the localized interaction microenvironment: the receptor protein is rendered as a light-green cartoon accompanied by a semi-transparent light-gray molecular surface; small-molecule ligands are shown as cyan or green sticks; key interacting amino acid residues are displayed as dark-blue sticks; and yellow dashed lines denote hydrogen bonds labeled with bond distances (in Å). Calculated binding affinities (kcal/mol) are indicated at the bottom of each panel. All structural models were visualized and rendered using PyMOL (v3.1.8).

## 4 Discussion

### 4.1 Overall clinical efficacy of SGLDQR formulas

Direct pairwise meta-analysis demonstrated that TCM formulas containing SGLDQR achieved a significantly higher overall clinical response rate than conventional WM in the non-surgical management of cholelithiasis [*RR* = 1.52, 95% *CI* (1.42, 1.63)], with negligible statistical heterogeneity (*I*² = 0.00%). Clinical characteristic evaluation across the WM nodes revealed that despite differences in the specific control regimens (UDCA, CDCA, and ANT), the trials exhibited sound consistency regarding dosing schedules, treatment durations, and outcome assessment criteria—primarily defined by symptom relief and stone reduction or expulsion. Subgroup analyses and univariate meta-regression further indicated that neither the category of control drug nor the duration of treatment exerted a significant effect modification on the overall pooled effect size (*P* > 0.05). In addition, sensitivity analyses restricted to networks using UDCA as the sole control confirmed that the effect sizes and relative hierarchical rankings of each herbal formulation remained robust. Collectively, the alignment between clinical plausibility and statistical verification establishes the clinical comparability of the control nodes, thereby firmly substantiating the transitivity assumption required for indirect comparisons within the model structure. The biological underpinnings of these macroscopic clinical benefits stem from the systematic modulation of the complex pathological cascade of cholelithiasis by TCM formulas. Conventional WM are largely restricted to unidimensional interventions: endogenous bile acid derivatives, such as UDCA and CDCA, primarily act by inhibiting intestinal cholesterol absorption and suppressing biliary cholesterol secretion, thereby reducing cholesterol saturation to achieve litholysis (35, 36). Meanwhile, the hydrocholeretic agent ANT facilitates stone clearance mainly through mechanical flushing by enhancing biliary motility and secretory function (37). In contrast to these single-target strategies, TCM formulas represented by SGLDQR demonstrate marked multi-target synergistic advantages. On the one hand, they restore gallbladder motility and facilitate stone expulsion by modulating the cholecystokinin (CCK) signaling pathway; on the other hand, they alleviate biliary inflammatory infiltration by downregulating pro-inflammatory cytokines such as TNF-α and IL-1β, thereby effectively mitigating concomitant symptoms including abdominal pain and diarrhea. Ultimately, these formulas achieve an integrative therapeutic regulation characterized by enhanced gallbladder evacuation and anti-inflammatory choleresis (38). In the NMA ranking, ZNDST exhibited the highest probability of clinical efficacy [*RR* = 2.08, 95% *CI* (1.39, 3.13); SUCRA = 88.2%]. From the perspective of formula compatibility, ZNDST adheres to the core therapeutic principle of “soothing the liver, clearing heat, and promoting choleresis.” By incorporating *Citri Reticulatae Pericarpium Viride* (Qingpi) to aggressively break qi stagnation and synergizing it with *Clematidis Radix et Rhizoma* (Weilingxian) and *Fritillariae Thunbergii Bulbus* (Zhebeimu) for their dispersing and nodule-resolving actions, the prescription embodies the classical strategy of “clearing heat and disinhibiting the gallbladder to treat the root cause, while circulating qi and resolving masses to address the symptoms.” This compatibility offers plausible clinical utility in alleviating biliary colic and facilitating the expulsion of microcalculi and biliary sludge. Modern pharmacological evidence provides micro-level mechanistic support: Weilingxian mitigates biliary congestion and promotes bile secretion via anti-inflammatory, analgesic, and choleretic pathways (39); Zhebeimu aids in softening and dissipating nodules to expedite stone excretion (40); and active constituents derived from Qingpi stimulate bile drainage through the regulation of bile acid transport-related cascades (41). Consequently, this multi-component synergy concurrently satisfies the multifaceted requirements of anti-inflammatory detumescence, choleretic litholysis/expulsion, and bile acid homeostasis restoration. It must be emphasized that the network ranking results are predominantly constrained by the star-shaped network geometry and the absence of closed loops for direct verification. Consequently, these rankings should be strictly regarded as exploratory and cannot be directly equated with established absolute clinical superiority. The prescription strategies derived from the respective formulas essentially represent hypothesis-generating models intended to guide personalized clinical adjustments rather than definitive treatment dogmas.

### 4.2 Heterogeneity analysis on TCM syndrome improvement and transaminase reduction

In the NMA of secondary outcomes, SUCRA values indicated that SGLDQRF conferred the highest probability of relative advantage in improving TCM syndrome scores (85.7%), whereas RPDSJN ranked first in reducing both AST (99.9%) and ALT (100.0%). Nonetheless, these hierarchies must be interpreted with extreme caution against the backdrop of substantial methodological and clinical heterogeneity, serving strictly as exploratory signals rather than definitive evidence of clinical superiority. Pairwise meta-analyses revealed profound statistical heterogeneity across these endpoints (TCM syndromes: *I*² = 87.3%; AST: *I*² = 99.1%; ALT: *I*² = 99.3%), paralleled by remarkably large between-study variances estimated within the Bayesian random-effects NMA framework (TCM syndromes: *τ*² = 1.936; AST: *τ*² = 525.206; ALT: *τ*² = 482.123). This heterogeneity stems largely from the scarce study volume (merely 3–4 RCTs per network) and pronounced clinical divergence among the included trials. On the one hand, patients in the RPDSJN trial presented with markedly elevated baseline transaminase levels (mean AST ≈ 150.00 U/L; ALT ≈ 170.00 U/L), whereas those in the SJLDT and SGLDQRF trials had baseline values strictly within physiological ranges (25–28 U/L), naturally precluding substantial downward modulation. Consequently, the prominent ranking of RPDSJN is intrinsically confounded by the mathematical artifact of regression toward normality in severe baseline populations. On the other hand, for TCM syndrome evaluations, baseline scores in the SGHST trial (11.20–12.04 points) were significantly lower than those in the remaining studies (16.75–18.95 points); furthermore, the intervention duration for ZNSGLDT was limited to 2 months compared to 6 months in other trials. This marked disparity in baseline disease severity and treatment exposure inevitably magnified the underlying statistical heterogeneity. From the perspectives of modern pharmacology and formula compatibility, the distinct therapeutic emphases of these formulas offer a biologically plausible rationale for the aforementioned clinical divergence. SGLDQRF represents an organic integration of Xiaochaihu Decoction and Sijin Decoction. Xiaochaihu Decoction focuses primarily on harmonizing the Shaoyang meridian; its active flavonoids modulate the TLR4/NF-*κ*B signaling pathway to confer hepatoprotective and anti-inflammatory benefits, thereby substantially reducing TCM syndrome scores (42, 43). Meanwhile, Sijin Decoction excels at clearing heat and promoting diuresis to facilitate lithagogue actions, reinforced by *Gigeriae Galli Endothelium Corneum* (Jineijin) to dissolve stones and resolve stagnation (44–46). The synergistic interplay among these constituent botanicals effectively alleviates biliary inflammatory spasms and dull abdominal pain (45–49), providing robust pharmacological underpinnings for the systematic improvement of comprehensive patient syndromes. In comparison, RPDSJN is formulated upon the structural backbone of Xiaoshi Fanshi Powder, supplemented with *Rhei Radix et Rhizoma* (Dahuang), *Paeoniae Radix Rubra* (Chishao), *Citri Reticulatae Pericarpium* (Chenpi), and *Evodiae Fructus* (Wuzhuyu). Within this formulation, *Sal Nitri* (Xiaoshi) and *Alumen* (Baifan) facilitate dampness elimination and jaundice resolution (50); emodin substantially improves biliary kinetics (51, 52); total glucosides of paeony effectively disrupt the oxidative stress cascade (53, 54); and Chenpi synergizes with Wuzhuyu to attenuate hepatocellular inflammatory infiltration (55, 56). Collectively, these multi-component actions exhibit marked hepatoprotective and transaminase-lowering potential, particularly in patients presenting with acute cholestatic liver injury. In summary, the stratified therapeutic paradigm proposed in this study—namely, favoring hepatoprotective and purgative formulas (e.g., RPDSJN) in cases with pronounced hepatic impairment, versus prioritizing harmonizing and clearing formulas (e.g., SGLDQRF) when systemic symptoms predominate alongside near-normal transaminase profiles—essentially constitutes a hypothesis-generating model aimed at guiding the clinical integration of disease diagnosis and syndrome differentiation. Its definitive therapeutic superiority warrants further verification through rigorously designed, high-quality clinical trials with well-balanced baseline characteristics.

### 4.3 Compatibility mechanisms against damp-heat and gallbladder qi stagnation

Pathologically, cholelithiasis originates in the liver and gallbladder but frequently affects the spleen and stomach. This aligns with the classic principle in the *Synopsis of Prescriptions of the Golden Chamber (Jingui Yaolve)*: “Seeing disease of the liver, one knows it will transmit to the spleen, and must therefore first fortify the spleen,” emphasizing the pathological progression across the liver, gallbladder, and spleen/stomach (57). Targeting “damp-heat accumulation and liver-gallbladder qi stagnation,” the herbal property-taste compatibility follows distinct regularities: bitter herbs (Huangqin, Dahuang) purge and dry damp-heat; pungent herbs (Chaihu, Yujin) disperse liver-gallbladder qi; and sweet herbs (Gancao, Baishao) strengthen the spleen, harmonize the stomach, soften the liver, and alleviate pain (58). The core herbs are predominantly cold/cool, echoing the principle in *Shennong's Classic of Materia Medica Commentary* (*Shennong Bencao Jing Shu*) that “yin-cold conquers heat” to collectively clear damp-heat (59). These key herbs primarily enter the liver, spleen, and stomach meridians, embodying simultaneous liver-soothing and spleen-strengthening effects. Therapeutically, “unblocking” (*Tong*) serves as the primary principle, supplemented by “eliminating” (*Xiao*) and “tonifying” (*Bu*) (60). To maximize gallbladder function through unblocking, the formulation strategically integrates three synergistic therapeutic dimensions based on the disease stage: (1) Damp-heat clearance and stone excretion: Jinqiancao and Yujin are combined to promote choleresis and expel stones; (2) Shaoyang harmonization and bowel purgation: Chaihu, Huangqin, and Dahuang clear damp-heat and descend bowel qi to drain the gallbladder, aligning with the classic doctrine of “purging the large intestine to drain the gallbladder”; and (3) Liver softening and spleen strengthening: Baishao,Gancao, and Jineijin dissolve stone accumulations while consolidating the constitution. Consequently, this multi-target integration achieves a holistic regulation that embodies the essence of “disease–syndrome combination.”

### 4.4 SGLDQR formulas modulate the immune–lipid crosstalk network in cholelithiasis

#### 4.4.1 Pathophysiological basis of the “immune–lipid crosstalk” in cholelithiasis

Cholelithiasis is increasingly conceptualized as a systemic immuno-metabolic disorder orchestrated by an “immune–lipid crosstalk” network, rather than an isolated localized biliary pathology (63). At the pathophysiological level, this regulatory network is collaboratively driven by two tightly coupled dimensions: localized mucosal inflammatory remodeling and systemic dyslipidemia. Within the local microenvironment, pro-inflammatory stimuli such as lipopolysaccharide (LPS) aberrantly activate the gallbladder mucosal TLR2/4–NF-*κ*B signaling axis. This cascade triggers both the hypersecretion of pro-nucleating mucins (MUC2 and MUC5AC) and the infiltration of neutrophils, which in turn assemble neutrophil extracellular traps (NETs). These NETs serve as an indispensable physical fibrillar scaffold that fosters cholesterol microcrystal nucleation and precipitation (61, 62). Concomitantly, localized oxidative stress induces gallbladder smooth muscle contractility impairment, thereby compounding biliary stasis (63). At the systemic level, dysregulated lipid metabolism disrupts the farnesoid X receptor (FXR) signaling axis, which not only drives biliary cholesterol supersaturation (manifested as an elevated cholesterol saturation index, CSI), but also markedly blunts the kinetic responsiveness of the biliary smooth muscle to cholecystokinin (CCK) (64). This self-perpetuating, bidirectional vicious cycle between local inflammatory responses and systemic metabolic derangement constitutes the core pathological driver governing lithogenesis, crystal aggregation, and sustained disease progression.

#### 4.4.2 Multi-target synergistic mechanisms and clinical efficacy mapping of SGLDQR TCM formulae

Based on network pharmacology and molecular docking analyses, the integrated clinical benefits elicited by SGLDQR TCM formulae—characterized by accelerated calculus expulsion rates, rapid symptom alleviation, and marked restoration of hepatic function—are fundamentally driven by multi-target synergistic regulation mediated by a core network of active compounds. This regulatory network predominantly consists of five pivotal bioactive constituents: the flavonoids quercetin (derived from *Desmodium styracifolium* and *Bupleurum chinense*), wogonin, and oroxylin A (both from *Scutellaria baicalensis*); the monoterpene glycoside paeoniflorin (derived from *Paeonia lactiflora*); and the anthraquinone aloe-emodin (derived from *Rheum officinale*/*Rheum palmatum*). Regarding biliary local microenvironment remodeling, the clinically observed alleviation of biliary colic and imaging-indicated clearance of microcalculi/biliary sludge may largely be attributed to the synergistic pharmacological actions between mucosal inflammation resolution and nucleation matrix scaffold destabilization: quercetin blocks the NOX2/ROS/NF-*κ*B signaling axis (65, 66); *wogonin* induces macrophage polarization toward the anti-inflammatory M2 phenotype via the LSD1–p65 axis (67); and oroxylin A contributes to restoring regulatory T cell/T helper 17 (Treg/Th17) immune homeostasis (68, 69). Synergistically with paeoniflorin, these monomers significantly attenuate LPS-mediated inflammatory cascades, downregulate mucin hypersecretion, and potentially disrupt the physical stone nucleation scaffolds formed by NETs (70); meanwhile, quercetin exhibits potential relaxing effects on biliary smooth muscle by inhibiting extracellular Ca²⁺ influx, thereby facilitating bile fluid dynamics and alleviating cholestasis (71). At the cellular and molecular levels, these coordinated pathways provide a plausible biological rationale for the clinically observed alleviation of biliary spasm and stone clearance benefits. At the level of systemic metabolic regulation, the laboratory-documented reduction in biliary CSI and the normalization of serum transaminase levels may plausibly correspond to the coordinated engagement of lipid metabolic modulation and hepatoprotective pathways: quercetin and oroxylin A alleviate hepatic lipid accumulation and pyroptosis via the IRS-2/PI3 K/Akt and AMPK/FGF21 signaling axes, respectively (72–74); wogonin activates the FXR to facilitate negative feedback regulation of bile acid excretion and promote CSI reduction (75); and paeoniflorin induces mitophagy via an Nrf2-dependent mechanism to ameliorate hepatic lipid metabolic dysregulation (76, 77). Crucially, these pharmacodynamic mechanisms are reinforced by synergistic spatiotemporal pharmacokinetics. Aloe-emodin inherently displays a natural hepatobiliary tropism. Co-existing components within the formula further enhance its bioavailability and target organ exposure by inhibiting efflux transporters and promoting intestinal absorption (78, 79). This pharmacokinetic–pharmacodynamic (PK–PD) synergy helps sustain therapeutic drug concentrations within target tissues, effectively translating the network pharmacology-predicted targets into tangible clinical benefits.

## 5 Limitations and future perspectives

The findings of this study should be interpreted in light of several limitations: (1) **Methodological quality:** Inadequate reporting on allocation concealment and blinding in primary trials raises the risk of detection and performance biases, especially for subjective TCM syndrome and clinical response scores. (2) **Network structure:** The star-shaped network lacked closed loops, preventing inconsistency testing and making indirect comparisons strictly reliant on transitivity; additionally, merging distinct Western regimens (UDCA, CDCA, and ANT) means SUCRA rankings remain exploratory rather than confirmatory. (3) **Heterogeneity:** Findings for TCM syndrome scores, AST, and ALT were derived from limited studies with marked heterogeneity, likely driven by baseline transaminase and duration differences, indicating potential advantages rather than established efficacy. (4) **Safety:** Sparse, passive, and short-term reporting of adverse events precludes definitive conclusions on comparative or long-term safety. (5) **Computational predictions:** Network pharmacology and molecular docking provide static, hypothesis-generating insights sensitive to database filtering, not direct mechanistic proof. (6) **Generalizability:** The single-region trial population warrants caution when extrapolating results to broader cohorts. Future work should prioritize multicenter, double-blind RCTs adhering to CONSORT standards with long-term follow-up, alongside *in vitro* and *in vivo* functional assays to validate predicted pathways.

## 6 Conclusion

This NMA suggests that SGLDQR TCM formulas offer potential clinical benefits in non-surgical cholelithiasis management compared with conventional WM. SUCRA values indicated exploratory trends: ZNDST ranked relatively highest for overall effective rate, SGLDQRF showed a potential advantage in improving TCM syndrome scores, and RPDSJN exhibited a regulatory tendency toward lowering AST/ALT levels. However, given the clinical heterogeneity of control regimens, substantial statistical heterogeneity and data sparsity for transaminases and syndrome scores, alongside baseline variations, these SUCRA rankings and the proposed syndrome-differentiation strategy remain exploratory hypotheses rather than established clinical superiority. Prescription data mining revealed that these formulations synergistically integrate heat-clearing, dampness-draining, and cholagogic principles, reflecting multitarget compatibility patterns. In silico predictions (network pharmacology and molecular docking) involving key active compounds (quercetin, paeoniflorin, and aloe-emodin) provide biological hypotheses targeting inflammation and lipid metabolism, rather than definitive causal evidence. Furthermore, sparse adverse event reporting precludes firm conclusions regarding comparative or long-term safety. Rigorously designed, multicenter double-blind RCTs with standardized regimens and long-term follow-up, paired with experimental biological validation, are warranted to confirm therapeutic efficacy, safety, and mechanisms.

## Data Availability

The original contributions presented in the study are included in the article/Supplementary Material, further inquiries can be directed to the corresponding author.
