# Supplementary material for: Clinical efficacy and multi-target mechanisms of liver-dispersing, bile-inducing, and heat-clearing TCM formulas in cholelithiasis: network meta-analysis, network pharmacology, and molecular docking study
Source: Front Med (Lausanne). 2026 Oct 7;13:1944318. doi: 10.3389/fmed.2026.1944318 (PMC13683209; doi:10.3389/fmed.2026.1944318)
Supplement: Supplementary file 1 [file Datasheet1.doc]

**Supplementary Materials**

**Supplementary Information, Figures, and Tables**

**Page 2: Information S-1.** Search strategies.

**Page 5: Figure S-1.** Sensitivity analysis of direct pairwise meta-analysis for clinical efficacy using the random-effects model.

**Page 5: Figure S-2.** Leave-one-out sensitivity analysis for clinical efficacy.

**Page 6: Figure S-3.** Top 20 significantly enriched Gene Ontology (GO) terms for the 87 candidate targets.

**Page 6: Figure S-4.** Top 20 significantly enriched Kyoto Encyclopedia of Genes and Genomes (KEGG) pathways for the 87 candidate targets.

**Page 7: Table S-1.** Diagnostic criteria sources for the 13 included studies.

**Page 8-9: Table S-2.** Baseline disease characteristics of the included studies.

**Page 9-10: Table S-3.** Summary of clinical efficacy assessment criteria in the included studies.

**Page 11-12: Table S-4.** Nomenclature, abbreviations, and herbal compositions of the 12 SGLDQR formulas.

**Page 12: Table S-5.** Summary of comparator regimens in the included studies.

**Page 12: Table S-6.** Subgroup analyses for clinical efficacy.

**Page 13: Table S-7.** Meta-regression results for clinical efficacy.

**Page 13: Table S-8.** Network meta-analysis results for clinical efficacy.

**Page 14: Table S-9.** Network meta-analysis results for clinical efficacy.

**Page 15: Table S-10.** Comparison of treatment rankings between main and sensitivity analyses.

**Page 15: Table S-11.** Baseline, post-treatment, and change-from-baseline TCM syndrome scores (points) across the four trials evaluated in the network meta-analysis.

**Page 16: Table S-12.** Pairwise league table for TCM syndrome scores (points) derived from the network meta-analysis.

**Page 16: Table S-13.** Baseline, post-treatment, and change-from-baseline serum AST levels (U/L) across the three trials evaluated in the network meta-analysis.

**Page 17: Table S-14.** Baseline, post-treatment, and change-from-baseline serum ALT levels (U/L) across the three trials evaluated in the network meta-analysis.

**Page 17: Table S-15.** Pairwise league table for serum AST levels (U/L) derived from the network meta-analysis.

**Page 18: Table S-16.** Pairwise league table for serum ALT levels (U/L) derived from the network meta-analysis.

**Page 19-21: Table S-17.** CINeMA quality assessment for clinical efficacy.

**Page 22: Table S-18.** Latin names with Chinese pinyin names of single herbs in included 13 studies.

**Page 23: Table S-19.** Frequent herb pairs derived from association rules with minimum support of 50% and confidence of 80%.

**Page 24-26: Table S-20.** List of active ingredients for pharmacological screening of herb-target networks.

**Page 27: Table S-21.** Nomenclature and corresponding protein details of key genes.

**Page 27: Table S-22.** Receptor structural parameters and docking grid box configurations.

**Information S-1. Search strategies**

From the date of establishing the database to December 31, 2024, the retrieval strategies for the eight databases are as follows:

**1. CNKI database：**

TI=(胆石症 + 胆总管结石病)AND AB=(随机对照试验 + 随机 + 对照 + 临床研究 + 临床试验 + 临床对照)AND TI=(中医 +	中西医 +	中药 + 中草药 +中成药 + 复方 + 法 +	自拟 +	氏 + 汤 + 方 + 散 + 丹 + 胶囊 + 丸 + 颗粒 + 剂)

**2. Wanfang database：**

题名:(胆石症 OR 胆总管结石病)AND 摘要:(随机对照试验 OR 随机 OR 对照OR 临床研究 OR 临床试验 OR 临床对照)AND 题名:(中医 OR	中西医 OR中药 OR 中草药 OR	中成药 OR 复方 OR 法 OR	自拟 OR	氏 OR 汤 OR

方 OR 散 OR 丹 OR 胶囊 OR 丸 OR 颗粒 OR 剂)

**3. VIP database：**

M=(胆石症 OR 胆总管结石病) AND R=(随机对照试验 OR 随机 OR 对照 OR临床研究 OR 临床试验 OR 临床对照) AND T=(中医 OR 中西医 OR 中药OR 中草药 OR 中成药 OR 复方 OR 法 OR	自拟 OR	氏 OR 汤 OR 方 OR散 OR 丹 OR 胶囊 OR 丸 OR 颗粒 OR 剂)

**4. CBM database:**

((((("汤"[标题:智能] OR "方"[标题:智能] OR "散"[标题:智能] OR "丹"[标题:智能] OR "胶囊"[标题:智能] OR " 丸"[标题:智能] OR "颗粒"[标题:智能] OR "剂"[标题:智能]) OR (("中医"[标题:智能] OR " 中西医"[标题:智能] OR " 中药"[标题:智能] OR " 中草药"[标题:智能] OR " 中成药"[标题:智能] OR " 复方"[标题:智能] OR "法"[标题:智能] OR " 自拟"[标题:智能] OR " 氏"[标题:智能]) AND (("胆石症"[标题:智能] OR "胆总管结石病"[标题:智能])AND (("随机对照试验"[摘要:智能] OR " 随机"[摘要:智能] OR "对照"[摘要:智能] OR " 临床研究"[摘要:智能] OR " 临床研究"[摘要:智能] OR "临床对照"[摘要:智能])

**5. Pumed:**

(（( "Cholelithiasis"[Mesh] ) OR "Choledocholithiasis"[Mesh]）OR ((((((Cholelithiases[Title/Abstract])) OR (Gallstone Disease[Title/Abstract])) OR (Gallstone Diseases[Title/Abstract])) OR (Cholelithiasis[Title/Abstract])) OR (Choledocholithiasis[Title/Abstract])) OR (Common Bile Duct[Title/Abstract])）AND ((("Medicine, Chinese Traditional"[Mesh]) OR (((((((((Chinese Medicine, Traditional[Title/Abstract]) OR (Chinese Traditional Medicine[Title/Abstract])) OR (Chung I Hsueh[Title/Abstract])) OR (Traditional Chinese Medicine[Title/Abstract])) OR (Traditional Medicine, Chinese[Title/Abstract])) OR (Traditional Tongue Assessment[Title/Abstract])) OR (Traditional Tongue Diagnosis[Title/Abstract])) OR (Zhong Yi Xue[Title/Abstract])) OR (TCM[Title/Abstract]))) OR (("Drugs, Chinese Herbal"[Mesh]) OR (((((((Chinese Drugs, Plant[Title/Abstract]) OR (Chinese Herbal Drugs[Title/Abstract])) OR (Plant Extracts, Chinese[Title/Abstract])) OR (Chinese Herbal[Title/Abstract])) OR (Tang[Title/Abstract])) OR (Fang[Title/Abstract])) OR (Prescription[Title/Abstract]))))) AND (((((((("Randomized Controlled Trials as Topic"[Mesh]) OR ("Randomized Controlled Trial" [Publication Type])) OR (RCT[Title/Abstract])) OR (Randomly[Title/Abstract])) OR (Randomized[Title/Abstract])) OR (Controlled Clinical Trial[Title/Abstract])) OR (Clinical Trial[Title/Abstract])) OR (Clinical Study[Title/Abstract])))

**6. Web of Science:**

TS=(Cholelithiasis OR Cholelithiases OR Gallstone Diseases OR Gallstone Disease OR Choledocholithiasis	OR Common Bile Duct	) AND TS=(Medicine, Chinese Traditional OR Chinese Medicine, Traditional OR Chinese Traditional Medicine OR Chung I Hsueh OR Traditional Chinese Medicine OR Traditional Medicine, Chinese OR Traditional Tongue Assessment OR Traditional Tongue Diagnosis OR Zhong Yi Xue OR TCM OR Drugs, Chinese Herbal OR Chinese Drugs, Plant OR Chinese Herbal Drugs OR Plant Extracts, Chinese OR Chinese Herbal OR Tang OR Fang OR Prescription) AND AB=(Randomized Controlled Trial OR RCT OR Randomly OR Randomized OR Controlled Clinical Trial OR Clinical Trial OR Clinical Study)

**7. The Cochrane Library:**

ID	Search

#1	MeSH descriptor: [Cholelithiasis] explode all trees

#2	(Cholelithiasis):ti,ab,kw OR (Gallstone Disease):ti,ab,kw OR (Gallstone Diseases):ti,ab,kw (Word variations have been searched)

#3	#1 OR #2

#4	MeSH descriptor: [Choledocholithiasis] explode all trees

#5	(Choledocholithiasis):ti,ab,kw OR (Common Bile Duct):ti,ab,kw AND (Cholelithiasis):ti,ab,kw (Word variations have been searched)

#6	#4 OR #5

#7	#3 OR #6

#8	MeSH descriptor: [Medicine, Chinese Traditional] explode all trees

#9	(Chinese Medicine, Traditional or Chinese Traditional Medicine or Chung I Hsueh or Traditional Chinese Medicine or Traditional Medicine, Chinese or Traditional Tongue Assessment or Traditional Tongue Diagnosis or Zhong Yi Xue or TCM):ti,ab,kw (Word variations have been searched)

#10 #8 OR #9

#11 MeSH descriptor: [Drugs, Chinese Herbal] explode all trees

#12 (Chinese Drugs, Plant or Chinese Herbal Drugs or Plant Extracts, Chinese or

Chinese Herbal or Tang or Fang or Prescription):ti,ab,kw (Word variations have been searched)

#13 #11 OR #12

#14 MeSH descriptor: [Randomized Controlled Trial] explode all trees

#15 (RCT or Randomly or Randomized or Controlled Clinical Trial or Clinical Trial or Clinical Study):ti,ab,kw (Word variations have been searched)

#16 #14 OR #15

#17 #7 AND #10 AND #13AND #16

**8. EMbase:**

#1 'cholelithiasis'/exp

#2 'common bile duct stone'/exp

#3 'bile calculosis':ab,ti OR 'bile lithiasis':ab,ti OR 'bile lithogenesis':ab,ti OR 'bile

lithogenicity':ab,ti OR 'biliary calculi':ab,ti OR 'biliary calculosis':ab,ti OR 'biliary

calculous disease':ab,ti OR 'biliary calculus':ab,ti OR 'biliary lithiasis':ab,ti OR 'biliary lithogenesis':ab,ti OR 'biliary lithogenicity':ab,ti OR 'biliary stone':ab,ti OR 'biliary

tract calculi':ab,ti OR 'biliary tract calculosis':ab,ti OR 'biliary tract calculous

disease':ab,ti OR 'biliary tract calculus':ab,ti OR 'biliary tract lithiasis':ab,ti OR 'biliary tract stone':ab,ti OR 'cholecystic lithiasis':ab,ti OR 'cholecysto lithiasis':ab,ti OR

cholecystolithiasis:ab,ti OR cholelith:ab,ti OR choleliths:ab,ti OR cholethiasis:ab,ti

OR 'gall bladder calculi':ab,ti OR 'gall bladder calculus':ab,ti OR 'gall bladder

concretions':ab,ti OR 'gall bladder lithiasis':ab,ti OR 'gall bladder stone':ab,ti OR

'gallbladder calculi':ab,ti OR 'gallbladder calculus':ab,ti OR 'gallbladder

concrements':ab,ti OR 'gallbladder concretion':ab,ti OR 'gallbladder lithiasis':ab,ti OR 'gallbladder stone':ab,ti OR 'gallstone disease':ab,ti

#4 'choledochal calculi':ab,ti OR 'choledochal calculus':ab,ti OR 'choledochal

stone':ab,ti OR choledocholithiasis:ab,ti OR 'choledochus calculi':ab,ti OR

'choledochus calculus':ab,ti OR 'choledochus stone':ab,ti OR 'common bile duct calculi':ab,ti OR 'common bile duct calculus':ab,ti OR 'common biliary duct

stone':ab,ti OR 'common duct gallstone':ab,ti OR 'ductus choledochus stone':ab,ti OR 'common bile duct stone':ab,ti

#5	#1 OR #2 OR #3 OR #4

#6 'chinese medicine'/exp

#7'chinese herbal medicine':ab,ti OR 'chinese traditional medicine':ab,ti OR 'medicine, chinese traditional':ab,ti OR 'traditional chinese medicine':ab,ti OR 'chinese

medicine':ab,ti OR tang:ab,ti OR fang:ab,ti OR prescription:ab,ti

#8	#6 OR #7

#9	'randomized controlled trial'/exp

#10	'randomized controlled trial':ab,ti OR rct:ab,ti OR randomly:ab,ti OR

randomized:ab,ti OR 'controlled clinical trial':ab,ti OR 'clinical trial':ab,ti OR 'clinical study':ab,ti

#11	#9 OR #10

#12	#5 AND #8 AND #11

**Figure S-1. Sensitivity analysis of direct pairwise meta-analysis for clinical efficacy using the random-effects model.**

**Figure S-2. Leave-one-out sensitivity analysis for clinical efficacy.**

**Figure S-3. Top 20 significantly enriched Gene Ontology (GO) terms for the 87 candidate targets.**

**Figure S-4. Top 20 significantly enriched Kyoto Encyclopedia of Genes and Genomes (KEGG) pathways for the 87 candidate targets.**

**Table S-1. Diagnostic criteria sources for the 13 included studies.**

| Study | (a) 1987 TCM Guideline | (b) 2002 TCM Guideline | (c) 2017 TCM Consensus | (d) Practice of Surgery | (e) 2018 GI Consensus | (f) Stone Disease Encyclopedia | (g) GI TCM Textbooks |
| --- | --- | --- | --- | --- | --- | --- | --- |
| Zhao & Liu, 2004 | ✓ |  |  |  |  |  |  |
| Xu, Chen & Pan, 2006 |  | ✓ |  |  |  |  |  |
| Guo, 2009 |  |  |  |  |  |  |  |
| Xing et al., 2009 |  |  |  |  |  | ✓ |  |
| Tan, 2010 |  | ✓ |  |  |  |  |  |
| Shi, 2012 |  | ✓ |  |  |  |  |  |
| Xu & Dang, 2013 |  |  |  |  |  |  |  |
| Dai et al., 2013 |  | ✓ |  |  |  |  |  |
| Guo, Zhao & Zhang, 2015 |  |  |  |  |  |  |  |
| Wang, 2019 |  | ✓ | ✓ | ✓ |  |  |  |
| Zhao, 2020 |  | ✓ | ✓ | ✓ | ✓ |  |  |
| Li et al., 2021 |  |  |  |  |  |  | ✓ |
| Yu, 2021 |  | ✓ | ✓ | ✓ |  |  |  |

**Note:** Diagnostic criteria sources (short forms): (a) Guiding Principles for Clinical Research of Acute Cholecystitis and Cholelithiasis with Traditional Chinese Medicine (1987); (b) Guiding Principles for Clinical Research of New Traditional Chinese Medicines (2002); (c) Consensus on the Integrated TCM-Western Medicine Diagnosis and Treatment of Cholelithiasis (2017); (d) Practice of Surgery, 4th ed. ; (e) Chinese Consensus on the Medical Management of Chronic Cholecystitis and Cholelithiasis (2018); (f) Encyclopedia of Diagnosis and Treatment of Stone Diseases (2000); (g) Clinical Diagnosis and Treatment of Digestive Diseases with TCM (2000) and Integrated TCM-Western Medicine Gastroenterology (2003). “✓” indicates that the study explicitly cited the corresponding diagnostic criteria; blank cells indicate not cited. Studies with no diagnostic criteria explicitly reported (Guo, 2009; Xu & Dang, 2013; Guo, Zhao & Zhang, 2015) are listed as blank across all columns and described in the text as "not reported."

**Table S-2. Baseline disease characteristics of the included studies.**

| **Study** | **Sample size (T/C)** | **Stone location** | | **Stone count** | | **Stone size** | | **Clinical stage** | |
| --- | --- | --- | --- | --- | --- | --- | --- | --- | --- |
|  |  | **T** | **C** | **T** | **C** | **T** | **C** | **T** | **C** |
| Zhao & Liu, 2004 | 100/100 | GB 83, CBD 7, IHD 10, GB+CBD 6, GB+IHD 5, GB+IHD+CBD 7 | GB 78, CBD 13, IHD 9, GB+CBD 8, GB+IHD 9, GB+IHD+CBD 3 | Mult 81, Sing 19 | Mult 79, Sing 21 | Max 5.3 cm; sludge | Max 4.8 cm; sludge | Rem | Rem |
| Xu, Chen & Pan, 2006 | 62/64 | GB 40, CBD 16, IHD 6 | GB 39, CBD 17, IHD 8 | Mult 46, Sing 16 | Mult 48, Sing 16 | X-ray positive: 10 cases | X-ray positive: 9 cases | - | - |
| Guo, 2009 | 30/30 | GB 22, CBD 2, IHD 2, GB+CBD 4 | GB 20, CBD 2, IHD 3, GB+CBD 5 | - | - | >1 cm:5; 0.5–1 cm:6; <0.5 cm:13; sludge:6 | >1 cm:7; 0.5–1 cm:8; <0.5 cm:11; sludge:4 | - | - |
| Tan, 2010 | 45/36 | GB 31, CBD 7, IHD 3, GB+IHD+CBD 4 | GB 21, CBD 7, IHD 4, GB+IHD+CBD 4 | - | - | Max 2.5×2.1 cm; sludge | Max 2.2×1.7 cm; sludge | - | - |
| Shi, 2012 | 44/38 | - | - | - | - | >1 cm:8; 0.5–1 cm:26; <0.5 cm:10 | >1 cm:7; 0.5–1 cm:22; <0.5 cm:9 | - | - |
| Xu & Dang, 2013 | 30/30 | GB 21, CBD 2, IHD 3, GB+CBD 4 | GB 22, CBD 3, IHD 2, GB+CBD 3 | - | - | <0.5 cm:14; 0.5–1 cm:6; >1 cm:4; sludge:6 | >1 cm:5; 0.5–1 cm:9; <0.5 cm:12; sludge:4 | - | - |
| Dai et al., 2013 | 55/45 | GB ^e^ > 3.4±1.0 mm, CBD ^e^ > 7.5±1.6 mm, IHD ^e^ > 4.2±1.0 mm | GB ^e^ 3.0±1.3 mm, CBD ^e^ 6.1±3.4 mm, IHD ^e^ 3.9±1.1 mm | - | - | (duct diameters) | (duct diameters) | Mild 15 / Mod 23 / Sev 17 | Mild 13 / Mod 20 / Sev 12 |
| Wang, 2019 | 50/49 | GB-predominant + CBD 1 case | GB-predominant + IHD 1 case | - | - | - | - | - | - |
| Zhao, 2020 | 49/49 | GB-predominant + CBD 2 cases | GB-predominant + CBD 1 case | - | - | - | - | - | - |
| Li et al., 2021 | 40/40 | - | - | 5.23±1.72 stones | 4.85±1.44 stones | 8.72±1.33 mm | 7.93±1.41 mm | - | - |

**Note:** T = experimental group; C = control group. GB = gallbladder; CBD = common bile duct; IHD = intrahepatic duct; "+" indicates concurrent stones in multiple locations. "-" = indicates no data available. "Mult" = multiple; "Sing" = solitary; "Rem" = remission phase; "Mod" = moderate; "Sev" = severe. In Dai et al. 2013, the values under GB/CBD/IHD represent duct diameters (mean ± SD, mm), not stone counts. Imaging modalities varied across studies (plain abdominal X-ray, B-ultrasound, etc.); data are presented as originally reported. Three studies (Xing et al., 2009; Guo, Zhao & Zhang, 2015; Yu, 2021) did not report stone-related baseline characteristics (e.g., location, number, or size of gallstones) and are therefore not presented in this table. All included studies reported that baseline characteristics were comparable between the treatment and control groups (all *P* > 0.05).

**Table S-3. Summary of clinical efficacy assessment criteria in the included studies.**

| **Study** | **Efficacy grade** | **Symptom-based criteria** | **Imaging-based criteria** |
| --- | --- | --- | --- |
| Zhao & Liu, 2004 | ① Resolution  ② Markedly effective  ③ Effective  ④ Ineffective | – | ① Complete disappearance of stones ② Reduction/shrinkage > 1/2 ③ Reduction/shrinkage ≤ 1/2 ④ No change |
| Xu, Chen & Pan, 2006 | ① Resolution  ② Markedly effective  ③ Effective  ④ Ineffective | Symptom score reduction:  ① > 95%  ② 70% to < 95%  ③ 30% to < 70%  ④ < 30% | ① Complete disappearance  ② Reduction in diameter > 0.5 cm or decrease > 1/3  ③ Reduction in diameter > 0.2 cm but ≤ 0.5 cm, or decrease < 1/3  ④ No change or enlargement |
| Guo, 2009 | ① Resolution  ② Effective  ③ Ineffective | ① Symptoms resolved  ② Symptoms alleviated  ③ No improvement | ① No stone shadows on B-ultrasound  ② Marked reduction in stone burden  ③ No change |
| Xing et al., 2009 | ① Resolution  ② Markedly effective  ③ Ineffective | ① Symptoms resolved  ② Symptoms/signs markedly alleviated  ③ No change | ① No stone shadows on B-ultrasound  ② Reduction/shrinkage > 50%  ③ No change |
| Tan, 2010 | ① Resolution  ② Improved  ③ Ineffective | ① Symptoms resolved  ② Symptoms/signs alleviated  ③ No change; or acute abdomen considered ineffective | ① No stone shadows on B-ultrasound  ② Reduction/shrinkage > 30%  ③ No change, or reduction/shrinkage < 30% |
| Shi, 2012 | ① Resolution  ② Markedly effective  ③ Effective  ④ Ineffective | – | ① Complete disappearance of stones ② Marked reduction/shrinkage > 1/2 ③ Reduction/shrinkage ≤ 1/2 ④ No change |
| Xu & Dang, 2013 | ① Resolution  ② Effective  ③ Ineffective | ① Symptoms resolved  ② Symptoms alleviated  ③ No improvement | ① No stone shadows on B-ultrasound  ② Marked reduction in stone burden  ③ No change |
| Dai et al., 2013 | ① Resolution  ② Markedly effective  ③ Effective  ④ Ineffective | Symptom score reduction:  ① ≥ 95%  ② ≥ 70%  ③ ≥ 30%  ④ < 30% | ① Complete disappearance  ② Reduction in diameter > 0.5 cm or decrease > 1/3  ③ Reduction in diameter > 0.2 cm but ≤ 0.5 cm, or decrease < 1/3  ④ No change or enlargement |
| Guo, Zhao & Zhang, 2015 | ① Resolution  ② Markedly effective  ③ Effective  ④ Ineffective | ① Symptoms resolved  ② Symptoms/signs markedly improved  ③ Symptoms/signs improved  ④ No improvement or deterioration | ① Complete disappearance of stones  ② Reduction/shrinkage > 1/2  ③ Reduction/shrinkage ≤ 1/2  ④ No change |
| Wang, 2019 | ① Resolution  ② Markedly effective  ③ Effective  ④ Ineffective | – | ① Complete disappearance of stones; morphology restored to normal  ② Bile duct dilatation relieved, or reduction in stone diameter > 0.5 cm  ③ Reduction in stone diameter ≤ 0.5 cm  ④ No change or enlargement |
| Zhao, 2020 | ① Resolution  ② Markedly effective  ③ Effective  ④ Ineffective | – | ① Complete disappearance of stones; morphology restored to normal ② Bile duct dilatation relieved, or reduction in stone diameter ≥ 50% ③ Reduction in stone diameter < 50% ④ No change or enlargement |
| Li et al., 2021 | ① Resolution  ② Markedly effective  ③ Effective  ④ Ineffective | – | ① Complete disappearance of biliary stones; morphology restored to normal  ② Bile duct dilatation relieved; reduction in stone diameter > 5 mm or decrease in number > 1/3  ③ Reduction in stone diameter < 5 mm or decrease in number < 1/3  ④ No change, or complications (e.g., impaction, infection, perforation) |
| Yu, 2021 | ① Resolution  ② Markedly effective  ③ Effective  ④ Ineffective | – | ① Complete disappearance of stones; morphology restored to normal  ② Reduction in stone diameter/size > 50%  ③ Reduction in stone diameter/size < 50%  ④ No change or enlargement |

**Note:** “–” indicates that symptom-based criteria were not explicitly reported in the original study (i.e., imaging-based criteria only). All efficacy grades are ordered from highest to lowest (i.e., Resolution > Markedly effective > Effective > Ineffective). Studies assessed by Zhao & Liu (2004) and Shi (2012) employed B-ultrasound plus one additional imaging modality (e.g., CT or MRCP). Wang (2019) and Zhao (2020) used B-ultrasound as the primary imaging tool, supplemented by CT or MRCP when necessary. Yu (2021) relied primarily on hepatobiliary color Doppler ultrasound. All criteria are presented as originally reported; the grading terms across different studies (e.g., “clinical cure” vs. “resolution”) have been standardized for consistency.

**Table S-4. Nomenclature, abbreviations, and herbal compositions of the 12 SGLDQR formulas.**

| **Nomenclature** | **Abbreviations** | **Herbal compositions** |
| --- | --- | --- |
| Rongpai Gallstone-Dissolving Capsule | RPDSJN | *Citri Reticulatae Pericarpium Viride, Citri Reticulatae Pericarpium, Aucklandiae Radix, Aurantii Fructus Immaturus, Curcumae Radix, Paeoniae Radix Rubra, Galli Gigerii Endothelium Corneum, Alumen, Natrii Sulfas, Rhei Radix et Rhizoma, Crataegi Fructus, Scrophulariae Radix, Glycyrrhizae Radix et Rhizoma, Artemisiae Scopariae Herba, Lonicerae Japonicae Flos, Lysimachiae Herba, Codonopsis Radix, Angelicae Sinensis Radix, Euodiae Fructus, Scutellariae Radix* |
| Modified Sini Powder | SNSJJ | *Bupleuri Radix, Paeoniae Radix Rubra, Paeoniae Radix Alba, Aurantii Fructus, Curcumae Radix, Lygodii Spora, Galli Gigerii Endothelium Corneum, Setariae Fructus Germinatus, Hordei Fructus Germinatus, Citri Sarcodactylis Fructus, Lysimachiae Herba, Polygoni Cuspidati Rhizoma et Radix, Rhei Radix et Rhizoma, Glycyrrhizae Radix et Rhizoma* |
| Gandan Shu Pill | GDSW | *Bupleuri Radix, Paeoniae Radix Alba, Aurantii Fructus, Toosendan Fructus, Corydalis Rhizoma, Curcumae Radix, Salviae Miltiorrhizae Radix et Rhizoma, Lysimachiae Herba, Magnoliae Officinalis Cortex, Aurantii Fructus Immaturus, Scutellariae Radix, Citri Reticulatae Pericarpium Viride, Cyperi Rhizoma, Aucklandiae Radix, Natrii Sulfas Exsiccatus, Rhei Radix et Rhizoma* |
| Jinzhi Huang Formula | JZHF | *Galli Gigerii Endothelium Corneum, Aurantii Fructus, Lysimachiae Herba, Paeoniae Radix Alba, Rhei Radix et Rhizoma, Corydalis Rhizoma, Glycyrrhizae Radix et Rhizoma* |
| Self-Designed Gallstone Decoction | ZNDST | *Bupleuri Radix, Aurantii Fructus, Menthae Haplocalycis Herba, Citri Reticulatae Pericarpium Viride, Clematidis Radix et Rhizoma, Fritillariae Thunbergii Bulbus, Remanniae Radix, Curcumae Radix* |
| Self-Designed Jinji Gallstone Decoction | ZNJJDST | *Lysimachiae Herba, Galli Gigerii Endothelium Corneum, Polygoni Cuspidati Rhizoma et Radix, Bupleuri Radix, Aurantii Fructus, Citri Reticulatae Pericarpium Viride, Cyperi Rhizoma, Artemisiae Scopariae Herba, Taraxaci Herba, Curcumae Radix, Corydalis Rhizoma* |
| Qinggan Lidan Paishi Pill | QGLDPSW | *Rubus ichangensis, Artemisiae Scopariae Herba, Lysimachiae Herba, Polygoni Cuspidati Rhizoma et Radix, Bupleuri Radix, Gardeniae Fructus, Rhei Radix et Rhizoma, Galli Gigerii Endothelium Corneum, Aucklandiae Radix, Aurantii Fructus Immaturus, Paeoniae Radix Alba, Glycyrrhizae Radix et Rhizoma* |
| Self-Designed Lidan Sanjin Decoction | ZNLDSJT | *Lysimachiae Herba, Galli Gigerii Endothelium Corneum, Curcumae Radix, Aucklandiae Radix, Coptidis Rhizoma, Bupleuri Radix, Magnoliae Officinalis Cortex, Paeoniae Radix Alba, Aurantii Fructus Immaturus, Glycyrrhizae Radix et Rhizoma* |
| Shugan Huashi Decoction | SGHST | *Bupleuri Radix, Pinelliae Rhizoma, Scutellariae Radix, Coptidis Rhizoma, Angelicae Sinensis Radix, Paeoniae Radix Alba, Atractylodis Macrocephalae Rhizoma, Poria, Codonopsis Radix, Curcumae Radix, Galli Gigerii Endothelium Corneum, Lygodii Spora, Lysimachiae Herba, Glycyrrhizae Radix et Rhizoma, Zingiberis Rhizoma Recens, Jujubae Fructus* |
| Sijin Lidan Decoction | SJLDT | *Bupleuri Radix, Scutellariae Radix, Coptidis Rhizoma, Curcumae Radix, Galli Gigerii Endothelium Corneum, Lygodii Spora, Lysimachiae Herba, Glycyrrhizae Radix et Rhizoma, Codonopsis Radix, Pinelliae Rhizoma, Poria, Corydalis Rhizoma, Atractylodis Macrocephalae Rhizoma, Paeoniae Radix Alba, Aucklandiae Radix, Zingiberis Rhizoma Recens, Jujubae Fructus* |
| Self-Designed Shugan Lidan Decoction | ZNSGLDT | *Lysimachiae Herba, Artemisiae Scopariae Herba, Bupleuri Radix, Scutellariae Radix, Curcumae Radix, Galli Gigerii Endothelium Corneum, Aurantii Fructus, Rhei Radix et Rhizoma, Citri Reticulatae Pericarpium Viride, Paeoniae Radix Alba, Polygoni Cuspidati Rhizoma et Radix, Toosendan Fructus, Glycyrrhizae Radix et Rhizoma* |
| Shugan Lidan Qingre Formula | SGLDQRF | *Bupleuri Radix, Pinelliae Rhizoma, Scutellariae Radix, Coptidis Rhizoma, Angelicae Sinensis Radix, Paeoniae Radix Alba, Artemisiae Scopariae Herba, Curcumae Radix, Lygodii Spora, Lysimachiae Herba, Galli Gigerii Endothelium Corneum, Glycyrrhizae Radix et Rhizoma, Zingiberis Rhizoma Recens, Jujubae Fructus* |

**Table S-5. Summary of comparator regimens in the included studies.**

| **Study** | **Comparator** | **Daily dose** |
| --- | --- | --- |
| Zhao & Liu, 2004 | UDCA tablets | 500mg |
| Xu, Chen & Pan, 2006 | UDCA capsules | 500-750mg |
| Guo, 2009 | ANT capsules | 75 mg ^a^ |
| Xing et al., 2009 | CDCA tablets | 1500 mg |
| Tan, 2010 | UDCA tablets | 8–10 mg/kg |
| Shi, 2012 | UDCA ^b^ | 600 mg |
| Xu & Dang, 2013 | ANT capsules | 75 mg ^a^ |
| Dai et al., 2013 | UDCA capsules | 500 mg |
| Guo, Zhao & Zhang, 2015 | UDCA capsules | 10–12 mg/kg |
| Wang, 2019 | UDCA capsules | 10 mg/kg |
| Zhao, 2020 | UDCA capsules | 10 mg/kg |
| Li et al., 2021 | UDCA capsules | 8–10 mg/kg |
| Yu, 2021 | UDCA capsules | 10 mg/kg |

**Note:** ^a^ Fixed-dose regimen (25 mg/tablet, three times daily); not weight-adjusted. ^b^ Reported only as "UDCA" in the original study; formulation (capsule or tablet) and manufacturer were not specified. All comparator drugs were administered orally. UDCA, ursodeoxycholic acid; CDCA, chenodeoxycholic acid; ANT, anetholtrithione.

**Table S-6. Subgroup analyses for clinical efficacy.**

| **Variables** |  | **N** | **Heterogeneity** | | **RR** | **(95% CI)** | **p-value** |
| --- | --- | --- | --- | --- | --- | --- | --- |
|  |  |  | **I^2^ (%)** | **p-value** |  |  |  |
| Total |  | 13 | 0.0% | 0.585 | 1.52 | (1.42,1.63) | <0.001 |
| Different western medicine controls | UDCA | 10 | 0.0% | 0.576 | 1.58 | (1.45,1.72) | <0.001 |
|  | ANT | 2 | 0.0% | 0.712 | 1.30 | (1.06,1.60) | 0.012 |
|  | CDCA | 1 | 0.0% | <0.001 | 1.42 | (1.21,1.67) | <0.001 |
| Treatment duration | 1–3 months | 7 | 8.1% | 0.367 | 1.50 | (1.37,1.66) | <0.001 |
|  | 4–6 months | 6 | 0.0% | 0.630 | 1.54 | (1.39,1.71) | <0.001 |

**Note:** UDCA, ursodeoxycholic acid; CDCA, chenodeoxycholic acid; ANT, anetholtrithione.

**Table S-7. Meta-regression results for clinical efficacy.**

| **Indicators** | **Covariate** | **N** | **Coef.** | **Std.Err.** | **t** | **p** | **95%CI** | |
| --- | --- | --- | --- | --- | --- | --- | --- | --- |
| Clinical efficacy | Different western medicine controls | 13 | -0.211 | 0.139 | -1.52 | 0.157 | -0.518 | 0.95 |
|  | Treatment duration | 13 | 0.044 | 0.197 | 0.23 | 0.826 | -0.389 | 0.478 |

**Table S-8. Network meta-analysis results for clinical efficacy.**

| **Intervention** | ***RR* [95% *CI*]** | | | | | | | | | | | | |
| --- | --- | --- | --- | --- | --- | --- | --- | --- | --- | --- | --- | --- | --- |
|  | **ZNDST** | **ZNSGLDT** | **QGLDPSW** | **RPDSJN** | **ZNJJDST** | **SNSJJ** | **SGHST** | **JZHF** | **ZNLDSJT** | **SJLDT** | **SGLDQRF** | **GDSW** | **WM** |
| ZNDST | 1 |  |  |  |  |  |  |  |  |  |  |  |  |
| ZNSGLDT | 1.04 [0.53, 2.04] | 1 |  |  |  |  |  |  |  |  |  |  |  |
| QGLDPSW | 1.20 [0.70, 2.08] | 1.16 [0.61, 2.22] | 1 |  |  |  |  |  |  |  |  |  |  |
| RPDSJN | 1.25 [0.81, 1.96] | 1.20 [0.69, 2.13] | 1.04 [0.70, 1.54] | 1 |  |  |  |  |  |  |  |  |  |
| ZNJJDST | 1.23 [0.72, 2.13] | 1.19 [0.62, 2.27] | 1.02 [0.61, 1.69] | 0.99 [0.66, 1.47] | 1 |  |  |  |  |  |  |  |  |
| SNSJJ | 1.35 [0.86, 2.13] | 1.30 [0.74, 2.33] | 1.12 [0.74, 1.69] | 1.08 [0.83, 1.41] | 1.10 [0.72, 1.67] | 1 |  |  |  |  |  |  |  |
| SGHST | 1.45 [0.89, 2.38] | 1.39 [0.76, 2.56] | 1.19 [0.76, 1.89] | 1.15 [0.83, 1.59] | 1.18 [0.74, 1.85] | 1.08 [0.76, 1.52] | 1 |  |  |  |  |  |  |
| JZHF | 1.47 [0.95, 2.27] | 1.41 [0.81, 2.44] | 1.20 [0.82, 1.79] | 1.16 [0.92, 1.47] | 1.18 [0.79, 1.75] | 1.08 [0.83, 1.41] | 1.01 [0.74, 1.39] | 1 |  |  |  |  |  |
| ZNLDSJT | 1.47 [0.94, 2.33] | 1.41 [0.80, 2.50] | 1.22 [0.81, 1.82] | 1.18 [0.90, 1.52] | 1.19 [0.78, 1.82] | 1.09 [0.82, 1.45] | 1.02 [0.72, 1.43] | 1.01 [0.78, 1.30] | 1 |  |  |  |  |
| SJLDT | 1.52 [0.93, 2.44] | 1.45 [0.79, 2.63] | 1.25 [0.79, 1.96] | 1.20 [0.87, 1.64] | 1.22 [0.78, 1.92] | 1.11 [0.79, 1.56] | 1.04 [0.71, 1.54] | 1.03 [0.75, 1.41] | 1.02 [0.74, 1.43] | 1 |  |  |  |
| SGLDQRF | 1.56 [0.97, 2.56] | 1.52 [0.83, 2.70] | 1.30 [0.83, 2.00] | 1.25 [0.92, 1.69] | 1.27 [0.81, 2.00] | 1.16 [0.83, 1.61] | 1.09 [0.74, 1.59] | 1.08 [0.79, 1.45] | 1.06 [0.77, 1.47] | 1.04 [0.72, 1.52] | 1 |  |  |
| GDSW | 1.61 [1.02, 2.50] | 1.54 [0.87, 2.70] | 1.32 [0.88, 2.00] | 1.27 [0.97, 1.67] | 1.30 [0.85, 1.96] | 1.18 [0.88, 1.59] | 1.10 [0.78, 1.56] | 1.09 [0.84, 1.41] | 1.09 [0.82, 1.45] | 1.06 [0.76, 1.49] | 1.02 [0.74, 1.41] | 1 |  |
| WM | 2.08 [1.39, 3.13] | 2.00 [1.16, 3.45] | 1.72 [1.20, 2.44] | 1.67 [1.39, 1.96] | 1.67 [1.16, 2.44] | 1.54 [1.25, 1.89] | 1.43 [1.09, 1.89] | 1.43 [1.20, 1.67] | 1.41 [1.16, 1.72] | 1.39 [1.05, 1.82] | 1.33 [1.02, 1.72] | 1.30 [1.06, 1.59] | 1 |

**Note:** Data are presented as risk ratios (*RRs*) with 95% confidence intervals (95% *CIs*). Effect estimates reflect the comparison of the row-defining intervention versus the column-defining intervention (Row vs. Column); an *RR* > 1.00 indicates higher clinical efficacy in favor of the row intervention. A 95% *CI* crossing the null value of 1.00 (e.g., 1.04 [0.53, 2.04]) indicates that the difference in efficacy between the two interventions is not statistically significant.

**Table S-9. Network meta-analysis results for clinical efficacy.**

| **Intervention** | ***RR* [95% *CI*]** | | | | | | | | | | |
| --- | --- | --- | --- | --- | --- | --- | --- | --- | --- | --- | --- |
|  | **ZNDST** | **ZNSGLDT** | **QGLDPSW** | **RPDSJN** | **ZNJJDST** | **SNSJJ** | **SGHST** | **ZNLDSJT** | **SJLDT** | **SGLDQRF** | **UDCA** |
| ZNDST | 1 |  |  |  |  |  |  |  |  |  |  |
| ZNSGLDT | 1.04 [0.53, 2.04] | 1 |  |  |  |  |  |  |  |  |  |
| QGLDPSW | 1.20 [0.70, 2.08] | 1.16 [0.61, 2.22] | 1 |  |  |  |  |  |  |  |  |
| RPDSJN | 1.25 [0.81, 1.96] | 1.20 [0.69, 2.13] | 1.04 [0.70, 1.54] | 1 |  |  |  |  |  |  |  |
| ZNJJDST | 1.23 [0.72, 2.13] | 1.19 [0.62, 2.27] | 1.02 [0.61, 1.69] | 0.99 [0.66, 1.47] | 1 |  |  |  |  |  |  |
| SNSJJ | 1.35 [0.86, 2.13] | 1.30 [0.74, 2.33] | 1.12 [0.74, 1.69] | 1.08 [0.83, 1.41] | 1.10 [0.72, 1.67] | 1 |  |  |  |  |  |
| SGHST | 1.45 [0.89, 2.38] | 1.39 [0.76, 2.56] | 1.19 [0.76, 1.89] | 1.15 [0.83, 1.59] | 1.18 [0.74, 1.85] | 1.08 [0.76, 1.52] | 1 |  |  |  |  |
| ZNLDSJT | 1.47 [0.94, 2.33] | 1.41 [0.80, 2.50] | 1.22 [0.81, 1.82] | 1.18 [0.90, 1.52] | 1.19 [0.78, 1.82] | 1.09 [0.82, 1.45] | 1.02 [0.72, 1.43] | 1 |  |  |  |
| SJLDT | 1.52 [0.93, 2.44] | 1.45 [0.79, 2.63] | 1.25 [0.79, 1.96] | 1.20 [0.87, 1.64] | 1.22 [0.78, 1.92] | 1.11 [0.79, 1.56] | 1.04 [0.71, 1.54] | 1.02 [0.74, 1.43] | 1 |  |  |
| SGLDQRF | 1.56 [0.97, 2.56] | 1.52 [0.83, 2.70] | 1.30 [0.83, 2.00] | 1.25 [0.92, 1.69] | 1.27 [0.81, 2.00] | 1.16 [0.83, 1.61] | 1.09 [0.74, 1.59] | 1.06 [0.77, 1.47] | 1.04 [0.72, 1.52] | 1 |  |
| UDCA | 2.08 [1.39, 3.13] | 2.00 [1.16, 3.45] | 1.72 [1.20, 2.44] | 1.67 [1.39, 1.96] | 1.67 [1.16, 2.44] | 1.54 [1.25, 1.89] | 1.43 [1.09, 1.89] | 1.41 [1.16, 1.72] | 1.39 [1.05, 1.82] | 1.33 [1.02, 1.72] | 1 |

**Note:** Data are presented as risk ratios (*RR*s) with 95% confidence intervals (95% *CI*s). Effect estimates reflect the comparison of the row-defining intervention versus the column-defining intervention (Row vs. Column); an *RR* > 1.00 indicates higher clinical efficacy in favor of the row intervention. A 95% *CI* crossing the null value of 1.00 (e.g., 1.04 [0.53, 2.04]) indicates that the difference in efficacy between the two interventions is not statistically significant.

**Table S-10. Comparison of treatment rankings between main and sensitivity analyses.**

| **Rank** | **Main analysis (WM as control)** | **Sensitivity analysis (UDCA as control)** | **Change** |
| --- | --- | --- | --- |
| **1** | ZNDST | ZNDST | **—** |
| **2** | ZNSGLDT | ZNSGLDT | **—** |
| **3** | QGLDPSW | QGLDPSW | **—** |
| **4** | RPDSJN | RPDSJN | **—** |
| **5** | ZNJJDST | ZNJJDST | **—** |
| **6** | SNSJJ | SNSJJ | **—** |
| **7** | SGHST | SGHST | **—** |
| **8** | JZHF | ZNLDSJT | **↑1** |
| **9** | ZNLDSJT | SJLDT | **↑1** |
| **10** | SJLDT | SGLDQRF | **↑1** |
| **11** | SGLDQRF | **—** | **—** |
| **12** | GDSW | **—** | **—** |

**Note:** Rank change reflects the rank shift from the primary analysis to the sensitivity analysis; "—" denotes no change in relative rank, and "↑" denotes rank advancement (the accompanying integer indicates the number of positions advanced). Note that this numerical advancement was purely mechanical due to the exclusion of higher-ranking interventions rather than an intrinsic improvement in therapeutic efficacy. The sensitivity analysis was restricted to trials employing UDCA as the active control. GDSW (controlled with anethole trithione, ANT) and JZHF (controlled with chenodeoxycholic acid, CDCA) were excluded due to differing comparator regimens. Following exclusion, the relative hierarchy of the remaining interventions (ZNDST > ZNSGLDT > QGLDPSW > RPDSJN > ZNJJDST > SNSJJ > SGHST > ZNLDSJT > SJLDT > SGLDQRF) remained completely identical to that in the primary network, demonstrating the methodological robustness of the primary findings. Detailed surface under the cumulative ranking curve (SUCRA) profiles and values are illustrated in Figure 5.

**Table S-11.Baseline, post-treatment, and change-from-baseline TCM syndrome scores (points) across the four trials evaluated in the network meta-analysis.**

| **Formula** | **Duration (months)** | **Intervention Group (TCM Formula)** | | | **Control Group (UDCA)** | | |
| --- | --- | --- | --- | --- | --- | --- | --- |
|  |  | **Baseline, Mean ± SD** | **Post-treatment, Mean ± SD** | **Change from Baseline, Mean ± SD** | **Baseline, Mean ± SD** | **Post-treatment, Mean ± SD** | **Change from Baseline, Mean ± SD** |
| SGHST | 6 | 11.20±2.37 | 4.26±1.92 | -6.94±2.18 | 12.04±2.79 | 6.49±2.00 | -5.55±2.49 |
| SJLDT | 6 | 18.06±3.96 | 6.96±2.44 | -11.10±3.46 | 17.51±4.08 | 9.71±3.19 | -7.80±3.72 |
| ZNSGLDT | 2 | 16.75±2.61 | 5.18±1.30 | -11.57±2.26 | 17.53±1.30 | 9.58±1.48 | -7.95±1.40 |
| SGLDQRF | 6 | 18.95±2.51 | 6.18±2.35 | -12.77±2.43 | 18.68±2.38 | 10.76±2.93 | -7.92±2.70 |

**Note:** TCM, Traditional Chinese Medicine; UDCA, ursodeoxycholic acid; SD, standard deviation. All evaluated trials exclusively utilized UDCA as the control regimen. Negative values for changes from baseline indicate a reduction in TCM syndrome scores, with larger negative values reflecting greater clinical symptom alleviation.

**Table S-12. Pairwise league table for TCM syndrome scores (points) derived from the network meta-analysis.**

| **Intervention** | ***MD* [95% *CI*]** | | | | |
| --- | --- | --- | --- | --- | --- |
|  | **SGLDQRF** | **ZNSGLDT** | **SJLDT** | **SGHST** | **UDCA** |
| SGLDQRF | 0 |  |  |  |  |
| ZNSGLDT | 1.23 [-2.88, 5.34 ] | 0 |  |  |  |
| SJLDT | 1.55 [-2.72, 5.82 ] | 0.32 [-3.87, 4.51 ] | 0 |  |  |
| SGHST | 3.46 [-0.67, 7.59 ] | 2.23 [-1.82, 6.28 ] | 1.91 [-2.30, 6.12 ] | 0 |  |
| UDCA | 4.85 [1.89, 7.81 ] | 3.62 [0.77, 6.47 ] | 3.30 [0.22, 6.38 ] | 1.39 [-1.49, 4.27 ] | 0 |

**Note:** Continuous outcomes were evaluated using a random-effects network meta-analysis model, with comparative effect estimates reported as mean differences (*MD*s) and 95% confidence intervals (95% *CI*s) in points. Effect sizes are estimated as the row-defining treatment versus the column-defining treatment (Row vs. Column). In the bottom row, estimates represent UDCA versus the respective TCM formulas; the positive values indicate greater score reductions in the TCM formula groups than in the UDCA group, which are numerically and statistically equivalent to the negative estimates (TCM formulas vs. UDCA) reported in the main text and Table 3. The overall between-study variance parameter (τ²) was estimated across trials and incorporated into the network to account for global heterogeneity. A 95% CI crossing the null value of 0.00 denotes no statistically significant difference between regimens. *MD*, mean difference; *CI*, confidence interval; TCM, Traditional Chinese Medicine; UDCA, ursodeoxycholic acid.

**Table S-13. Baseline, post-treatment, and change-from-baseline serum AST levels (U/L) across the three trials evaluated in the network meta-analysis.**

| **Formula** | **Duration (months)** | **Intervention Group (TCM Formula)** | | | **Control Group (UDCA)** | | |
| --- | --- | --- | --- | --- | --- | --- | --- |
|  |  | **Baseline, Mean ± SD** | **Post-treatment, Mean ± SD** | **Change from Baseline, Mean ± SD** | **Baseline, Mean ± SD** | **Post-treatment, Mean ± SD** | **Change from Baseline, Mean ± SD** |
| RPDSJN | 6 | 145.00±46.40 | 46.00±14.30 | -99.00±41.16 | 153.00±51.60 | 155.00±55.10 | 2.00±53.44 |
| SJLDT | 6 | 28.65±9.99 | 26.24±8.83 | -2.41±9.46 | 27.74±9.89 | 25.67±8.81 | -2.07±9.40 |
| SGLDQRF | 6 | 26.20±2.65 | 24.50±6.47 | -1.70±5.63 | 25.70±2.76 | 24.10±6.55 | -1.60±5.70 |

**Note:** AST, aspartate aminotransferase; TCM, Traditional Chinese Medicine; UDCA, ursodeoxycholic acid; SD, standard deviation. All evaluated trials exclusively employed UDCA as the control regimen. Negative values for change from baseline denote enzymatic reductions, with larger absolute negative values reflecting greater biochemical improvement in hepatic function.

Physiological reference range: According to a multicenter study conducted by the International Federation of Clinical Chemistry and Laboratory Medicine (IFCC) (Ceriotti et al., 2010), sex-related differences in the upper limit of normal (ULN) for serum AST were not clinically significant (differing by only 1.7 U/L); therefore, a single physiological reference interval of 11–34 U/L is established for healthy adults.

Reference: Ceriotti F, Henny J, Queraltó J, Ziyu S, Özarda Y, Chen B, et al. Common reference intervals for aspartate aminotransferase (AST), alanine aminotransferase (ALT) and γ-glutamyl transferase (GGT) in serum: results from an IFCC multicenter study. *Clin Chem Lab Med* (2010) 48:1593–1601. doi: 10.1515/CCLM.2010.315

**Table S-14. Baseline, post-treatment, and change-from-baseline serum ALT levels (U/L) across the three trials evaluated in the network meta-analysis.**

| **Formula** | **Duration (months)** | **Intervention Group (TCM Formula)** | | | **Control Group (UDCA)** | | |
| --- | --- | --- | --- | --- | --- | --- | --- |
|  |  | **Baseline, Mean ± SD** | **Post-treatment, Mean ± SD** | **Change from Baseline, Mean ± SD** | **Baseline, Mean ± SD** | **Post-treatment, Mean ± SD** | **Change from Baseline, Mean ± SD** |
| RPDSJN | 6 | 186.00±57.10 | 58.00±18.60 | -128.00±50.44 | 167.00±53.70 | 170.00±59.40 | 3.00±56.77 |
| SJLDT | 6 | 26.13±8.85 | 23.02±6.75 | -3.11±8.01 | 25.65±7.62 | 24.38±7.10 | -1.27±7.37 |
| SGLDQRF | 6 | 26.60±4.04 | 25.60±5.69 | -1.00±5.07 | 28.10±4.03 | 26.90±3.62 | -1.20±3.84 |

**Note:** ALT, alanine aminotransferase; TCM, Traditional Chinese Medicine; UDCA, ursodeoxycholic acid; SD, standard deviation. All evaluated trials exclusively employed UDCA as the control regimen. Negative values for change from baseline denote enzymatic reductions, with larger absolute negative values reflecting greater biochemical alleviation of hepatocellular injury.

Physiological reference range: According to a multicenter study conducted by the International Federation of Clinical Chemistry and Laboratory Medicine (IFCC) (Ceriotti et al., 2010), regional differences were not statistically significant; however, marked sex differences warrant partitioned reference limits. The physiological reference intervals are defined as 8–41 U/L for females and 9–59 U/L for males.

Reference: Ceriotti F, Henny J, Queraltó J, Ziyu S, Özarda Y, Chen B, et al. Common reference intervals for aspartate aminotransferase (AST), alanine aminotransferase (ALT) and γ-glutamyl transferase (GGT) in serum: results from an IFCC multicenter study. *Clin Chem Lab Med* (2010) 48:1593–1601. doi: 10.1515/CCLM.2010.315

**Table S-15. Pairwise league table for serum AST levels (U/L) derived from the network meta-analysis.**

| **Intervention** | ***MD* [95% *CI*]** | | | |
| --- | --- | --- | --- | --- |
|  | **RPDSJN** | **SGLDQRF** | **SJLDT** | **UDCA** |
| RPDSJN | 0 |  |  |  |
| SGLDQRF | 100.90 [35.97, 165.83] | 0 |  |  |
| SJLDT | 100.66 [35.67, 165.65] | -0.24 [-63.92, 63.44] | 0 |  |
| UDCA | 101.00 [54.18, 147.82] | 0.10 [-44.89, 45.09] | 0.34 [-44.73, 45.41] | 0 |

**Note:** AST, aspartate aminotransferase; *CI*, confidence interval; *MD*, mean difference; UDCA, ursodeoxycholic acid. Correspondence with Table 5: Following the standard "row versus column" (row minus column) convention, bottom-row comparisons of UDCA relative to TCM formulas yield positive estimates (e.g., UDCA vs. RPDSJN: *MD* = 101.00 U/L, 95% *CI* [54.18, 147.82]), where positive values indicate higher enzyme levels with UDCA (i.e., favoring TCM). These estimates are statistically equivalent and share identical absolute values with the negative estimates reported in Table 5 ("TCM minus UDCA"; *MD* = −101.00 U/L, 95% *CI* [−147.82, −54.18]).

**Table S-16. Pairwise league table for serum ALT levels (U/L) derived from the network meta-analysis.**

| **Intervention** | ***MD* [95% *CI*]** | | | |
| --- | --- | --- | --- | --- |
|  | **RPDSJN** | **SJLDT** | **SGLDQRF** | **UDCA** |
| RPDSJN | 0 |  |  |  |
| SJLDT | 129.16 [66.43, 191.89] | 0 |  |  |
| SGLDQRF | 131.20 [68.51, 193.89] | 2.04 [-58.93, 63.01] | 0 |  |
| UDCA | 131.00 [85.46, 176.54] | 1.84 [-41.30, 44.98] | -0.20 [-43.28, 42.88] | 0 |

**Notes:** ALT, alanine aminotransferase; CI, confidence interval; MD, mean difference; UDCA, ursodeoxycholic acid. Correspondence with Table 6: In accordance with the standard league-table convention of "row versus column" (row minus column), positive estimates in the bottom row denote higher enzyme levels with UDCA (i.e., favoring the TCM formula; e.g., UDCA vs. RPDSJN: *MD* = 131.00 U/L, 95% *CI* [85.46, 176.54]). These estimates share identical absolute values and complete statistical equivalence with the values reported under the reversed direction ("TCM minus UDCA") in Table 6 (e.g., RPDSJN vs. UDCA: *MD* = −131.00 U/L, 95% *CI* [−176.54, −85.46]), with opposing signs resulting solely from the inverted reference orientation.

**Table S-17. CINeMA quality assessment for clinical efficacy.**

| **Comparison** | **Within-study bias** | **Reporting bias** | **Indirectness** | **Imprecision** | **Heterogeneity** | **Incoherence** | **Confidence rating** |
| --- | --- | --- | --- | --- | --- | --- | --- |
| GDSW:WM | Some concerns | Some concerns | No concerns | No concerns | No concerns | No concerns | Low |
| JZHF:WM | Some concerns | Some concerns | No concerns | No concerns | No concerns | No concerns | Low |
| QGLDPSW:WM | Some concerns | Some concerns | No concerns | No concerns | No concerns | No concerns | Low |
| RPDSJN:WM | Some concerns | Some concerns | No concerns | No concerns | No concerns | No concerns | Low |
| SGHST:WM | Some concerns | Some concerns | No concerns | No concerns | No concerns | No concerns | Low |
| SGLDQRF:WM | Some concerns | Some concerns | No concerns | No concerns | No concerns | No concerns | Low |
| SJLDT:WM | Some concerns | Some concerns | No concerns | No concerns | No concerns | No concerns | Low |
| SNSJJ:WM | Some concerns | Some concerns | No concerns | No concerns | No concerns | No concerns | Low |
| ZNDST:WM | Some concerns | Some concerns | No concerns | No concerns | No concerns | No concerns | Low |
| ZNJJDST:WM | Some concerns | Some concerns | No concerns | No concerns | No concerns | No concerns | Low |
| ZNLDSJT:WM | Some concerns | Some concerns | No concerns | No concerns | No concerns | No concerns | Low |
| ZNSGLDT:WM | Some concerns | Some concerns | No concerns | No concerns | No concerns | No concerns | Low |
| GDSW:JZHF | Some concerns | Some concerns | No concerns | Some concerns | No concerns | No concerns | Very Low |
| GDSW:QGLDPSW | Some concerns | Some concerns | No concerns | Some concerns | No concerns | No concerns | Very Low |
| GDSW:RPDSJN | Some concerns | Some concerns | No concerns | Some concerns | No concerns | No concerns | Very Low |
| GDSW:SGHST | Some concerns | Some concerns | No concerns | Major concerns | No concerns | No concerns | Very Low |
| GDSW:SGLDQRF | Some concerns | Some concerns | No concerns | Major concerns | No concerns | No concerns | Very Low |
| GDSW:SJLDT | Some concerns | Some concerns | No concerns | Major concerns | No concerns | No concerns | Very Low |
| GDSW:SNSJJ | Some concerns | Some concerns | No concerns | Some concerns | No concerns | No concerns | Very Low |
| GDSW:ZNDST | Some concerns | Some concerns | No concerns | No concerns | No concerns | No concerns | Low |
| GDSW:ZNJJDST | Some concerns | Some concerns | No concerns | Some concerns | No concerns | No concerns | Very Low |
| GDSW:ZNLDSJT | Some concerns | Some concerns | No concerns | Some concerns | No concerns | No concerns | Very Low |
| GDSW:ZNSGLDT | Some concerns | Some concerns | No concerns | Some concerns | No concerns | No concerns | Very Low |
| JZHF:QGLDPSW | Some concerns | Some concerns | No concerns | Some concerns | No concerns | No concerns | Very Low |
| JZHF:RPDSJN | Some concerns | Some concerns | No concerns | Some concerns | No concerns | No concerns | Very Low |
| JZHF:SGHST | Some concerns | Some concerns | No concerns | Major concerns | No concerns | No concerns | Very Low |
| JZHF:SGLDQRF | Some concerns | Some concerns | No concerns | Major concerns | No concerns | No concerns | Very Low |
| JZHF:SJLDT | Some concerns | Some concerns | No concerns | Major concerns | No concerns | No concerns | Very Low |
| JZHF:SNSJJ | Some concerns | Some concerns | No concerns | Some concerns | No concerns | No concerns | Very Low |
| JZHF:ZNDST | Some concerns | Some concerns | No concerns | Some concerns | No concerns | No concerns | Very Low |
| JZHF:ZNJJDST | Some concerns | Some concerns | No concerns | Major concerns | No concerns | No concerns | Very Low |
| JZHF:ZNLDSJT | Some concerns | Some concerns | No concerns | Major concerns | No concerns | No concerns | Very Low |
| JZHF:ZNSGLDT | Some concerns | Some concerns | No concerns | Some concerns | No concerns | No concerns | Very Low |
| QGLDPSW:RPDSJN | Some concerns | Some concerns | No concerns | Major concerns | No concerns | No concerns | Very Low |
| QGLDPSW:SGHST | Some concerns | Some concerns | No concerns | Major concerns | No concerns | No concerns | Very Low |
| QGLDPSW:SGLDQRF | Some concerns | Some concerns | No concerns | Some concerns | No concerns | No concerns | Very Low |
| QGLDPSW:SJLDT | Some concerns | Some concerns | No concerns | Major concerns | No concerns | No concerns | Very Low |
| QGLDPSW:SNSJJ | Some concerns | Some concerns | No concerns | Major concerns | No concerns | No concerns | Very Low |
| QGLDPSW:ZNDST | Some concerns | Some concerns | No concerns | Major concerns | No concerns | No concerns | Very Low |
| QGLDPSW:ZNJJDST | Some concerns | Some concerns | No concerns | Major concerns | No concerns | No concerns | Very Low |
| QGLDPSW:ZNLDSJT | Some concerns | Some concerns | No concerns | Some concerns | No concerns | No concerns | Very Low |
| QGLDPSW:ZNSGLDT | Some concerns | Some concerns | No concerns | Major concerns | No concerns | No concerns | Very Low |
| RPDSJN:SGHST | Some concerns | Some concerns | No concerns | Some concerns | No concerns | No concerns | Very Low |
| RPDSJN:SGLDQRF | Some concerns | Some concerns | No concerns | Some concerns | No concerns | No concerns | Very Low |
| RPDSJN:SJLDT | Some concerns | Some concerns | No concerns | Some concerns | No concerns | No concerns | Very Low |
| RPDSJN:SNSJJ | Some concerns | Some concerns | No concerns | Some concerns | No concerns | No concerns | Very Low |
| RPDSJN:ZNDST | Some concerns | Some concerns | No concerns | Some concerns | No concerns | No concerns | Very Low |
| RPDSJN:ZNJJDST | Some concerns | Some concerns | No concerns | Major concerns | No concerns | No concerns | Very Low |
| RPDSJN:ZNLDSJT | Some concerns | Some concerns | No concerns | Some concerns | No concerns | No concerns | Very Low |
| RPDSJN:ZNSGLDT | Some concerns | Some concerns | No concerns | Major concerns | No concerns | No concerns | Very Low |
| SGHST:SGLDQRF | Some concerns | Some concerns | No concerns | Major concerns | No concerns | No concerns | Very Low |
| SGHST:SJLDT | Some concerns | Some concerns | No concerns | Major concerns | No concerns | No concerns | Very Low |
| SGHST:SNSJJ | Some concerns | Some concerns | No concerns | Major concerns | No concerns | No concerns | Very Low |
| SGHST:ZNDST | Some concerns | Some concerns | No concerns | Some concerns | No concerns | No concerns | Very Low |
| SGHST:ZNJJDST | Some concerns | Some concerns | No concerns | Major concerns | No concerns | No concerns | Very Low |
| SGHST:ZNLDSJT | Some concerns | Some concerns | No concerns | Major concerns | No concerns | No concerns | Very Low |
| SGHST:ZNSGLDT | Some concerns | Some concerns | No concerns | Major concerns | No concerns | No concerns | Very Low |
| SGLDQRF:SJLDT | Some concerns | Some concerns | No concerns | Major concerns | No concerns | No concerns | Very Low |
| SGLDQRF:SNSJJ | Some concerns | Some concerns | No concerns | Some concerns | No concerns | No concerns | Very Low |
| SGLDQRF:ZNDST | Some concerns | Some concerns | No concerns | Some concerns | No concerns | No concerns | Very Low |
| SGLDQRF:ZNJJDST | Some concerns | Some concerns | No concerns | Some concerns | No concerns | No concerns | Very Low |
| SGLDQRF:ZNLDSJT | Some concerns | Some concerns | No concerns | Major concerns | No concerns | No concerns | Very Low |
| SGLDQRF:ZNSGLDT | Some concerns | Some concerns | No concerns | Some concerns | No concerns | No concerns | Very Low |
| SJLDT:SNSJJ | Some concerns | Some concerns | No concerns | Major concerns | No concerns | No concerns | Very Low |
| SJLDT:ZNDST | Some concerns | Some concerns | No concerns | Some concerns | No concerns | No concerns | Very Low |
| SJLDT:ZNJJDST | Some concerns | Some concerns | No concerns | Major concerns | No concerns | No concerns | Very Low |
| SJLDT:ZNLDSJT | Some concerns | Some concerns | No concerns | Major concerns | No concerns | No concerns | Very Low |
| SJLDT:ZNSGLDT | Some concerns | Some concerns | No concerns | Major concerns | No concerns | No concerns | Very Low |
| SNSJJ:ZNDST | Some concerns | Some concerns | No concerns | Some concerns | No concerns | No concerns | Very Low |
| SNSJJ:ZNJJDST | Some concerns | Some concerns | No concerns | Major concerns | No concerns | No concerns | Very Low |
| SNSJJ:ZNLDSJT | Some concerns | Some concerns | No concerns | Some concerns | No concerns | No concerns | Very Low |
| SNSJJ:ZNSGLDT | Some concerns | Some concerns | No concerns | Major concerns | No concerns | No concerns | Very Low |
| ZNDST:ZNJJDST | Some concerns | Some concerns | No concerns | Major concerns | No concerns | No concerns | Very Low |
| ZNDST:ZNLDSJT | Some concerns | Some concerns | No concerns | Some concerns | No concerns | No concerns | Very Low |
| ZNDST:ZNSGLDT | Some concerns | Some concerns | No concerns | Major concerns | No concerns | No concerns | Very Low |
| ZNJJDST:ZNLDSJT | Some concerns | Some concerns | No concerns | Major concerns | No concerns | No concerns | Very Low |
| ZNJJDST:ZNSGLDT | Some concerns | Some concerns | No concerns | Major concerns | No concerns | No concerns | Very Low |
| ZNLDSJT:ZNSGLDT | Some concerns | Some concerns | No concerns | Major concerns | No concerns | No concerns | Very Low |

**Note:** Yellow, some concerns; Green, no concerns; Red, major concerns; Gold, low; Pink, very low.

**Table S-18. Latin names with Chinese pinyin names of single herbs in included 13 studies.**

| **No.** | **Latin names** | **Chinese pinyin names (Abbreviations)** |
| --- | --- | --- |
| 1 | *Lysimachiae Herba* | Jinqiancao(JQC) |
| 2 | *Curcumae Radix* | Yujin(YJ) |
| 3 | *Bupleuri Radix* | Chaihu(CH) |
| 4 | *Galli Gigerii Endothelium Corneum* | Jineijin(JNJ) |
| 5 | *Paeoniae Radix Alba* | Baishao(BS) |
| 6 | *Glycyrrhizae Radix et Rhizoma* | Gancao(GC) |
| 7 | *Rhei Radix et Rhizoma* | Dahuang(DH) |
| 8 | *Aurantii Fructus* | Zhike(ZK) |
| 9 | *Scutellariae Radix* | Huangqin(HQ) |
| 10 | *Citri Reticulatae Pericarpium Viride* | Qingpi(QP) |
| 11 | *Aurantii Fructus Immaturus* | Zhishi(ZS) |
| 12 | *Aucklandiae Radix* | Muxiang(MX) |
| 13 | *Artemisiae Scopariae Herba* | Yinchen(YC) |
| 14 | *Corydalis Rhizoma* | Yanhusuo(YHS) |
| 15 | *Coptidis Rhizoma* | Huanglian(HL) |
| 16 | *Polygoni Cuspidati Rhizoma et Radix* | Huzhang(HZ) |
| 17 | *Lygodii Spora* | Haijinsha(HJS) |
| 18 | *Pinelliae Rhizoma* | Banxia(BX) |
| 19 | *Zingiberis Rhizoma Recens* | Shengjiang(SJ) |
| 20 | *Poria* | Fuling(FL) |
| 21 | *Codonopsis Radix* | Dangshen(DS) |
| 22 | *Paeoniae Radix Rubra* | Chishao(CS) |
| 23 | *Magnoliae Officinalis Cortex* | Houpo(HP) |
| 24 | *Angelicae Sinensis Radix* | Danggui(DG) |
| 25 | *Jujubae Fructus* | Dazao(DZ) |
| 26 | *Cyperi Rhizoma* | Xiangfu(XF) |
| 27 | *Toosendan Fructus* | Chuanlianzi(CLZ) |
| 28 | *Natrii Sulfas Exsiccatus* | Xuanmingfen(XMF) |
| 29 | *Salviae Miltiorrhizae Radix et Rhizoma* | Danshen(DS) |
| 30 | *Alumen* | Baifan(BF) |
| 31 | *Atractylodis Macrocephalae Rhizoma* | Baizhu(BZ) |
| 32 | *Natrii Sulfas* | Mangxiao(MX) |
| 33 | *Euodiae Fructus* | Wuzhuyu(WZY) |
| 34 | *Clematidis Radix et Rhizoma* | Weilingxian(WLX) |
| 35 | *Scrophulariae Radix* | Xuanshen |
| 36 | *Rehmanniae Radix* | Shengdihuang(SDH) |
| 37 | *Lonicerae Japonicae Flos* | Jinyinhua(JYH) |
| 38 | *Setariae Fructus Germinatus* | Guya(GY) |
| 39 | *Taraxaci Herba* | Pugongying(PGY) |
| 40 | *Hordei Fructus Germinatus* | Maiya(MY) |
| 41 | *Citri Sarcodactylis Fructus* | Foshou(FS) |
| 42 | *Gardeniae Fructus* | Zhizi（ZZ） |
| 43 | *Rubus ichangensis Hemsl et Ktze* | Suanpaogen(SPG) |
| 44 | *Menthae Haplocalycis Herba* | Bohe(BH) |
| 45 | *Fritillariae Thunbergii Bulbus* | Zhebeimu(ZBM) |
| 46 | *Citri Reticulatae Pericarpium* | Chenpi(CP) |
| 47 | *Crataegi Fructus* | Shanzha(SZ) |

**Table S-19. Frequent herb pairs derived from association rules with minimum support of 50% and confidence of 80%.**

| **No** | **Lhs** | **Rhs** | **Support %** | **Confidence %** | **Lift** | **Count** |
| --- | --- | --- | --- | --- | --- | --- |
| 1 | *Galli Gigerii Endothelium Corneum* | *Lysimachiae Herba* | 77% | 100% | 1.08 | 10 |
| 2 | *Bupleuri Radix* | *Curcumae Radix* | 77% | 91% | 1.08 | 10 |
| 3 | *Bupleuri Radix* | *Lysimachiae Herba* | 77% | 91% | 0.99 | 10 |
| 4 | *Curcumae Radix* | *Bupleuri Radix* | 77% | 91% | 1.08 | 10 |
| 5 | *Curcumae Radix* | *Lysimachiae Herba* | 77% | 91% | 0.99 | 10 |
| 6 | *Lysimachiae Herba* | *Galli Gigerii Endothelium Corneum* | 77% | 83% | 1.08 | 10 |
| 7 | *Lysimachiae Herba* | *Bupleuri Radix* | 77% | 83% | 0.98 | 10 |
| 8 | *Lysimachiae Herba* | *Curcumae Radix* | 77% | 83% | 0.98 | 10 |
| 9 | *Lysimachiae Herba* | *Paeoniae Radix Alba* | 77% | 83% | 1.08 | 10 |
| 10 | *Paeoniae Radix Alba* | *Lysimachiae Herba* | 77% | 100% | 1.08 | 10 |
| 11 | *Galli Gigerii Endothelium Corneum* | *Glycyrrhizae Radix et Rhizoma* | 69% | 90% | 1.3 | 9 |
| 12 | *Bupleuri Radix* | *Paeoniae Radix Alba* | 69% | 82% | 1.07 | 9 |
| 13 | *Paeoniae Radix Alba* | *Bupleuri Radix* | 69% | 90% | 1.06 | 9 |
| 14 | *Glycyrrhizae Radix et Rhizoma* | *Galli Gigerii Endothelium Corneum* | 69% | 100% | 1.3 | 9 |
| 15 | *Glycyrrhizae Radix et Rhizoma* | *Lysimachiae Herba* | 69% | 100% | 1.08 | 9 |
| 16 | *Galli Gigerii Endothelium Corneum* | *Bupleuri Radix* | 62% | 80% | 0.95 | 8 |
| 17 | *Galli Gigerii Endothelium Corneum* | *Curcumae Radix* | 62% | 80% | 0.95 | 8 |
| 18 | *Galli Gigerii Endothelium Corneum* | *Paeoniae Radix Alba* | 62% | 80% | 1.04 | 8 |
| 19 | *Paeoniae Radix Alba* | *Galli Gigerii Endothelium Corneum* | 62% | 80% | 1.04 | 8 |
| 20 | *Paeoniae Radix Alba* | *Curcumae Radix* | 62% | 80% | 0.95 | 8 |
| 21 | *Paeoniae Radix Alba* | *Glycyrrhizae Radix et Rhizoma* | 62% | 80% | 1.16 | 8 |
| 22 | *Glycyrrhizae Radix et Rhizoma* | *Paeoniae Radix Alba* | 62% | 89% | 1.16 | 8 |
| 23 | *Scutellariae Radix* | *Curcumae Radix* | 54% | 100% | 1.18 | 7 |
| 24 | *Scutellariae Radix* | *Lysimachiae Herba* | 54% | 100% | 1.08 | 7 |
| 25 | *Rhei Radix et Rhizoma* | *Lysimachiae Herba* | 54% | 100% | 1.08 | 7 |

**Note:** Lhs, Left-Hand Side (antecedent herb); Rhs, Right-Hand Side (consequent herb). Support indicates the joint probability of both herbs appearing in the same prescription; Confidence represents the conditional probability of prescribing the Rhs herb given the presence of the Lhs herb.

**Table S-20. List of active ingredients for pharmacological screening of herb-target networks.**

| **Herbs** | **Number of the active ingredient** | **Molecule name** | **Herbs** | **Number of the active ingredient** | **Molecule name** | **Herbs** | **Number of the active ingredient** | **Molecule name** |
| --- | --- | --- | --- | --- | --- | --- | --- | --- |
| BS | BS1 | paeoniflorin | DH | DH1 | EUPATIN | HQ | HQ1 | wogonin |
|  | BS2 | paeoniflorgenone |  | DH2 | (-)-catechin |  | HQ2 | (2R)-7-hydroxy-5-methoxy-2-phenylchroman-4-one |
|  | BS3 | (3S,5R,8R,9R,10S,14S)-3,17-dihydroxy-4,4,8,10,14-pentamethyl-2,3,5,6,7,9-hexahydro-1H-cyclopenta[a]phenanthrene-15,16-dione |  | DH3 | Physciondiglucoside |  | HQ3 | baicalein |
|  | BS4 | Mairin |  | DH4 | aloe-emodin |  | HQ5 | 5,7,2,5-tetrahydroxy-8,6-dimethoxyflavone |
|  | BS5 | (+)-catechin |  | DH5 | rhein |  | HQ6 | Carthamidin |
|  | BS6 | Benzoic Acid |  | DH6 | Torachrysone-8-O-beta-D-(6'-oxayl)-glucoside |  | HQ7 | Dihydrobaicalin_qt |
|  | BS7 | Alexandrin,Daucosterol,Eleutheroside A |  | DH7 | Toralactone |  | HQ8 | Eriodyctiol (flavanone) |
|  | BS8 | Alexandrin,Daucosterol,Caproic Acid,Eleutheroside A,Sitogluside,Strumaroside,Î’-Sitosterol-Î’-D-Glucoside |  | DH8 | Emodin-1-O-beta-D-glucopyranoside |  | HQ9 | Salvigenin |
|  | BS9 | 2,3-O-(S)-Hexahydroxydiphenoyl-D-Glucopyranose |  | DH9 | Daucosterol_qt |  | HQ10 | 5,2',6'-Trihydroxy-7,8-dimethoxyflavone |
|  | BS10 | Paeonol |  | DH10 | Emodin |  | HQ11 | 5,7,2',6'-Tetrahydroxyflavone |
|  | BS11 | Scopoletin |  | DH11 | Chrysophanol |  | HQ12 | Skullcapflavone II |
|  | BS12 | Palbinone |  | DH12 | Physcion-8-Î’-D-Glucopyranoside |  | HQ13 | oroxylin a |
|  | BS13 | Aminobutyl Canavalmine |  | DH13 | Chrysarobin |  | HQ14 | Panicolin |
|  | BS14 | Canavalia Gibberellin I |  | DH14 | Chrysophanol-9-Anthrone |  | HQ15 | 5,7,4'-Trihydroxy-8-methoxyflavone |
| CH | CH1 | Linoleyl acetate |  | DH15 | Piceatannol |  | HQ16 | NEOBAICALEIN |
|  | CH2 | 3,5,6,7-tetramethoxy-2-(3,4,5-trimethoxyphenyl)chromone |  | DH16 | Torachrysone8-O-Î’-D-Glucopyranoside |  | HQ17 | DIHYDROOROXYLIN |
|  | CH3 | Areapillin |  | DH17 | 2-O-Cinnamoyl-Î’-Glucose |  | HQ18 | Norwogonin |
|  | CH4 | Cubebin |  | DH18 | Sennosidec |  | HQ19 | 5,2'-Dihydroxy-6,7,8-trimethoxyflavone |
|  | CH5 | Longikaurin A |  | DH19 | Sennosided |  | HQ20 | coptisine |
|  | CH6 | α-spinasterol |  | DH20 | Sennidinb |  | HQ21 | bis[(2S)-2-ethylhexyl] benzene-1,2-dicarboxylate |
|  | CH7 | petunidin |  | DH21 | Aloeemodin-Î©-O-Î’-D-Glucopyranoside |  | HQ22 | Diop |
|  | CH8 | (+)-Anomalin |  | DH22 | (+)-Catechin-5-O-Glucoside |  | HQ23 | epiberberine |
|  | CH9 | Cetylic Acid |  | DH23 | Chrysophanol-8-O-Î’-D-Glucopyranoside |  | HQ24 | Moslosooflavone |
|  | CH10 | Hexadecanoic Acid |  | DH24 | Chrysophanol-1-O-Î’-D-Glucoside |  | HQ25 | 11,13-Eicosadienoic acid, methyl ester |
|  | CH11 | Palmitic Acid |  | DH25 | 2-Cinnamoyl-Glucose |  | HQ26 | 5,7,4'-trihydroxy-6-methoxyflavanone |
|  | CH12 | Linolenic Acid |  | DH26 | Emodin-1-O-Î’-D-Glucopyranoside |  | HQ27 | 5,7,4'-trihydroxy-8-methoxyflavanone |
|  | CH13 | Octadecanoic?Acid |  | DH27 | (X{2212})-Epicatechin-3-O-Gallate |  | HQ28 | rivularin |
|  | CH14 | Stearic Acid |  | DH28 | 6-O-Galloyl-Glucose |  | HQ29 | Cetylic Acid,Hexadecanoic Acid,Palmitic Acid |
|  | CH15 | Caprylic Acid |  | DH29 | Palmidin C |  | HQ30 | Sucrose |
|  | CH16 | Isoquercitrin |  | DH30 | Physcion-8-O-Î’-D-Gentiobioside |  | HQ31 | Campesterol,M-Cresol |
|  | CH17 | Isoquercetrin |  | DH31 | Physcion-8-O-Î’-D-Glucopyranoside |  | HQ32 | 4'-Hydroxywogonin,5,7-Dihydroxy-8-Methoxylflavone |
|  | CH18 | Kuwanon H |  | DH32 | Piceid |  | HQ33 | Scutellarin |
|  | CH19 | Î‘-Spinasterol |  | DH33 | Procyanidin B1 3'-O-Gallate |  | HQ34 | Wogonin |
|  | CH20 | Caproic Acid |  | DH34 | Procyanidin B2 3,3'-Di-O-Gallate |  | HQ35 | 5-Hydroxy-6,7,8,4'-Tetramethoxyflavone,Skullcapflavone I,Skullcapflavone II |
|  | CH21 | Geraniol |  | DH35 | Resveratrol-4'-O-Î’-D-Glucoside |  | HQ36 | Oroxylin A |
|  | CH22 | Sabinene Hydrate |  | DH36 | Rheidin A |  | HQ37 | Skullcapflavone I |
|  | CH23 | Lignoceric Acid |  | DH37 | Rheidin B |  | HQ38 | Chrysin |
|  | CH24 | Enanthic Acid |  | DH38 | Rhein-8-O-Î’-D-Glucopyranoside |  | HQ39 | Isoschaftoside |
|  | CH25 | Eugenol |  | DH39 | Rheinoside A |  | HQ40 | 6-C-Arabinopyranosyl-8-C-Glucopyranosyl-5,7-Dihydroxyflavone |
|  | CH26 | Guaiacol |  | DH40 | Rheinoside B |  | HQ41 | 8-C-Arabinopyranosyl-6-C-Glucopyranosyl-5,7-Dihydroxyflavone |
|  | CH27 | 5-Hydroxycoumarin |  | DH41 | Rheinoside C |  | HQ42 | Baicalein |
|  | CH28 | Nonanoic Acid |  | DH42 | Rheinoside D |  | HQ43 | Baicalein-7-O-Î’-D-Glucopyranoside |
|  | CH29 | Petroselaidic Acid |  | DH43 | Rhein-8-O-Î’-D-(6'-Oxalyl)-Glucopyra-Noside |  | HQ44 | Dihydrooroxylin A |
|  | CH30 | Petroselinic Acid |  | DH44 | Sennidin A |  | HQ45 | 5,8-Dihydroxy-6,7-Dimethoxyflavone |
|  | CH31 | Saikochromoside A |  | DH45 | Sennoside A |  | HQ46 | 5,7-Dihydroxy-6,8,2',3'-Tetramethoxyflavone |
|  | CH32 | Saikosaponin A |  | DH46 | Sennoside C |  | HQ47 | 5,2'-Dihydroxy-6,7,8-Trimethoxyflavone |
|  | CH33 | Saikosaponin C |  | DH47 | 3,4,3',5'-Tetrahydroxystilbene-3-Glucoside |  | HQ48 | Eriodictyol |
|  | CH34 | Saikosaponin D |  | DH48 | Anthraglycoside B |  | HQ49 | 5-Hydroxy-7,8-Dimethoxyflavone |
|  | CH35 | Saikosaponin Q1 |  | DH49 | 5-Carboxy-7-Glucosyloxy-2-Methyl-Benzopyran-Î“-One |  | HQ50 | Isoscutellarein |
|  | CH36 | Saikosaponin Q2 |  | DH50 | 6-Hydroxymusizin-8-O-Î’-D-Glucoside |  | HQ51 | 7-Methoxybaicalein |
|  | CH37 | Saikosaponin S1 |  | DH51 | Musizin-8-O-Î’-D-Glucoside |  | HQ52 | 8-Methoxy-5-O-Glucoside Flavone |
|  | CH38 | Saikosaponin T |  | DH52 | Sennoside B |  | HQ53 | Norwogonin |
|  | CH39 | Saikosaponin V |  | DH53 | Torachrysone-8-O-Î’-D-Glucoside |  | HQ54 | 3,5,7,2'6'-Pentahydroxyflavanone |
|  | CH40 | Saikosaponin V1 |  | DH54 | Torachrysone-8-O-Î’-D-(6'-Oxayl)-Glucoside |  | HQ55 | 3,5,7,2',6'-Pentahydroxy Flavone |
|  | CH41 | Saikosaponin V2 | JQC | JQC1 | Linarin |  | HQ56 | Scutevulin |
|  | CH42 | 16Î‘,23,28,30-Tetrahydroxyolean-11,13(18)-Dien-3Î’-Yl-Î’-D-Glucopyranosyl-(1â†’3)-Î’-D-Fucopyranoside |  | JQC2 | Hesperetin |  | HQ57 | 5,7,2',5'-Tetrahydroxy-8,6'-Dimethoxy Flavone |
|  | CH43 | Thymol |  | JQC3 | daucostero_qt |  | HQ58 | 5,7,2',3'-Tetrahydroxyflavone |
| YJ | YJ1 | naringenin |  | JQC4 | rhamnocitrin-3,4'-diglucOside |  | HQ59 | 5,7,2',6'-Tetrahydroxyflavone |
|  | YJ2 | Camphor |  | JQC5 | Menthol |  | HQ60 | 2,6,2',4'-Tetrahydroxy-6â€™-Methoxychalcone |
|  | YJ3 | Curcumin |  | JQC6 | Citronellol |  | HQ61 | 5,2',6'-Trihydroxy-7,8-Dimethoxyflavone |
|  | YJ4 | P-Tolyl-Methyl Carbinol Diferuloyl Methane | BS, CH | C1 | Phenol |  | HQ62 | 5,7,2'-Trihydroxy-8,6'-Dimethoxyflavone |
| YJ, BS, DH, HQ | A1 | beta-sitosterol | BS, HQ | D1 | Sitosterol,Î’-Sitosterol |  | HQ63 | 5,8,2'-Trihydroxy-6,7-Dimethoxyflavone |
| CH, JQC, BS, DH | A2 | kaempferol | YJ, JQC, BS, HQ | D2 | sitosterol |  | HQ64 | 5,7,2'-Trihydroxyflavone |
| DH, BS | A3 | 1-O-Galloyl-Glucose | JQC, HQ | E1 | acacetin |  | HQ65 | 5,7,4'-Trihydroxy-6-C-Glucoside-8-C-Arabinoside Flavone |
| DH, BS | A4 | 3,4,5-Trihydroxybenzoic Acid,Gallic Acid,Gallicacidmonohydrate | JQC, HQ | E2 | ent-Epicatechin |  | HQ66 | 7,2',6'-Trihydroxy-5-Methoxychalcone |
| CH, HQ | B1 | Baicalin | JQC, CH | F1 | isorhamnetin |  | HQ67 | 5,7,4'-Trihydroxy-6-Methoxyflavanone |
| CH, HQ | B2 | Stigmasterol | JQC, CH | F2 | quercetin |  | HQ68 | 7,2'6'-Trihydroxy-5-Methoxyflavanone |
|  |  |  |  |  |  |  | HQ69 | 5,7,2'-Trihydroxy-6-Methoxyflavone |
|  |  |  |  |  |  |  | HQ70 | 5,8,2'-Trihydroxy-7-Methoxyflavone |
|  |  |  |  |  |  |  | HQ71 | 5,2',5'-Trihydroxy-6,7,8-Trimethoxyflavone |
|  |  |  |  |  |  |  | HQ72 | Viscidulin Iii-6'-O-Î’-D-Glucopyranoside |
|  |  |  |  |  |  |  | HQ73 | Wogonin 5-Î’-D-Glucoside |

**Table S-21. Nomenclature and corresponding protein details of key genes.**

| **Gene Symbol** | **Standard Protein Abbr.** | **Full Protein Name** |
| --- | --- | --- |
| *IL6* | IL-6 | Interleukin-6 |
| *IL1B* | IL-1β | Interleukin-1 beta |
| *TNF* | TNF-α | Tumor necrosis factor alpha |
| *IL10* | IL-10 | Interleukin-10 |
| *IFNG* | IFN-γ | Interferon gamma |
| *EGFR* | EGFR | Epidermal growth factor receptor |
| *CCL2* | CCL2 | C-C motif chemokine ligand 2 |
| *TLR4* | TLR4 | Toll-like receptor 4 |
| *ALB* | ALB | Serum albumin |
| *CXCL8* | CXCL8 | C-X-C motif chemokine ligand 8 |

**Table S-22. Receptor structural parameters and docking grid box configurations.**

| **Parameter** | **Target: IL-6** | **Target: IL-1β** | **Target: TNF-α** |
| --- | --- | --- | --- |
| Gene Symbol | *IL6* | *IL1B* | *TNF* |
| Receptor Complex Details | Structure of interleukin 6 (gp130 P496L mutant) | Structure of the IL-1b signaling complex | TNFR1 selectve TNF mutant; R1-6 |
| PDB ID | 8QY6 | 4DEP | 2ZJC |
| Chain | D | B | B |
| Method (Resolution) | Cryo-EM (3.16 Å) | X-ray (3.10 Å) | X-ray (2.50 Å) |
| Reference Ligand | NAG | NAG | GOL |
| Grid Box Center  (x, y, z) | (213.93, 226.15, 243.001) | (95.157, 59.876, 60.55) | (-9.552, 26.04, 5.542) |
| Grid Box Size  (x, y, z) (Å) | (100, 100, 100) | (100, 100, 100) | (60, 60, 60) |
| Exhaustiveness | 8 | 8 | 8 |
